# Modeling the impact of *Plasmodium falciparum* sexual stage immunity on the composition and dynamics of the human infectious reservoir for malaria in natural settings

**DOI:** 10.1371/journal.ppat.1007034

**Published:** 2018-05-09

**Authors:** André Lin Ouédraogo, Philip A. Eckhoff, Adrian J. F. Luty, Will Roeffen, Robert W. Sauerwein, Teun Bousema, Edward A. Wenger

**Affiliations:** 1 Institute for Disease Modeling, Intellectual Ventures, Bellevue, Washington, United States of America; 2 Département de Sciences Biomédicales, Centre National de Recherche et de Formation sur le Paludisme, Ouagadougou, Burkina Faso; 3 Department of Medical Microbiology, Radboud University Medical Center, Nijmegen, HB, the Netherlands; 4 MERIT UMR 216/CERPAGE, Institut de Recherche pour le Développement, Cotonou, Bénin; 5 UMR 216, Mère et enfant face aux infections tropicales, Institut de Recherche pour le Développement, Paris, France; Case Western Reserve University, UNITED STATES

## Abstract

Malaria transmission remains high in Sub-Saharan Africa despite large-scale implementation of malaria control interventions. A comprehensive understanding of the transmissibility of infections to mosquitoes may guide the design of more effective transmission reducing strategies. The impact of *P*. *falciparum* sexual stage immunity on the infectious reservoir for malaria has never been studied in natural settings. Repeated measurements were carried out at start-wet, peak-wet and dry season, and provided data on antibody responses against gametocyte/gamete antigens Pfs48/45 and Pfs230 as anti-gametocyte immunity. Data on high and low-density infections and their infectiousness to anopheline mosquitoes were obtained using quantitative molecular methods and mosquito feeding assays, respectively. An event-driven model for *P*. *falciparum* sexual stage immunity was developed and fit to data using an agent based malaria model infrastructure. We found that Pfs48/45 and Pfs230 antibody densities increased with increasing concurrent gametocyte densities; associated with 55–70% reduction in oocyst intensity and achieved up to 44% reduction in proportions of infected mosquitoes. We showed that *P*. *falciparum* sexual stage immunity significantly reduces transmission of microscopic (*p* < 0.001) but not submicroscopic (*p* = 0.937) gametocyte infections to mosquitoes and that incorporating sexual stage immunity into mathematical models had a considerable impact on the contribution of different age groups to the infectious reservoir of malaria. Human antibody responses to gametocyte antigens are likely to be dependent on recent and concurrent high-density gametocyte exposure and have a pronounced impact on the likelihood of onward transmission of microscopic gametocyte densities compared to low density infections. Our mathematical simulations indicate that anti-gametocyte immunity is an important factor for predicting and understanding the composition and dynamics of the human infectious reservoir for malaria.

## Author summary

Submicroscopic gametocyte infections are efficiently transmitted from humans to mosquitoes in settings with efficient malaria vectors and may pose challenges for malaria control and elimination efforts. Our understanding of what mechanisms contribute to submicroscopic gametocytes infectiousness remains limited. Here we assess the impact of naturally acquired anti-gametocyte antibodies on malaria transmission to mosquitoes and on the age-dependent composition of the infectious reservoir and seasonal dynamics. Anti-gametocyte immunity significantly reduces the infectiousness of high gametocyte density infections, contributes to explain the age-related profiles of the infectious reservoir in the study area, whilst submicroscopic gametocyte infections that present with lower anti-Pfs48/45 and anti-Pfs230 antibody responses commonly remain transmissible to mosquitoes. Our findings indicate that sexual stage immunity needs to be incorporated in transmission models to better understand transmission dynamics. Furthermore, tools that boost sexual stage immunity may reduce transmission to mosquitoes and thus aid elimination strategies.

## Introduction

The 2007 call issued by Bill and Melinda Gates for malaria eradication [1] has rapidly gained momentum. Malaria has plummeted globally and numerous countries continue to make significant progress toward elimination [2–5]. Nevertheless, more than a dozen countries in Sub-Saharan Africa, representing approximately 80% of the remaining global malaria burden, still need to considerably improve malaria control interventions substantially to reach levels of transmission that are suitable for elimination strategies [6]. In these countries where climate [7] and ecological conditions favor the vector’s life cycle, malaria transmission remains high and is likely compounded by under-resourced health care systems, resilient vector populations, and, obligatory for ongoing transmission, a reservoir of infectious individuals [8–11]. Although prioritizing vector control, early diagnosis and treatment of symptomatic malaria currently remain the foundation of malaria control, actual assessment and management of the human infectious reservoir may accelerate transmission reduction in highly endemic countries and contribute to sustaining elimination progress where transmission is low [12, 13].

Considerable efforts in diagnostics have been made to improve malaria case management and elimination strategies. More sensitive molecular methods now detect previously undiagnosed low-density infections [14–16] while point-of-care diagnostics such as rapid diagnostic tests play an important role in case management and may contribute considerably to elimination strategies once their sensitivity is improved [17]. Parasite detection or quantification form imperfect proxies for the infectious reservoir of malaria that can currently only be assessed by mosquito feeding assays [18–20].

*P*. *falciparum* sexual stage immunity may restrict parasite development within the mosquito’s midgut [21–25], and may thus affect the composition and dynamics of the infectious reservoir that is not only dictated by parasite or gametocyte densities [26]. The infectious reservoir for malaria negatively associates with age [11, 27, 28], with conflicting evidence on the contribution of submicroscopic infections to transmission [11, 29–33]. The impact of sexual stage immunity on the human infectious reservoir has never been studied and is not incorporated in mathematical models that play an increasingly important role in campaign strategies and policy making decisions [17, 34–36].

Naturally acquired sexual stage immunity is predominantly antibody-mediated rather than cellular [37, 38]. Antibodies against antigens that are shared between gametocytes and gametes are acquired upon natural gametocyte exposure and, once ingested as part of the mosquito blood meal, may interact with gamete surface-expressed antigens such as Pfs48/45 and Pfs230 [24, 39–45] and thereby reduce or completely block human-to-mosquito transmission. Functional sexual stage immunity, transmission reducing activity (TRA), can be estimated using an *in vitro* standard membrane feeding assay (SMFA) [46] in which a mixture of cultured gametocytes and purified antibodies is fed to mosquitoes. However, the SMFA may only detect the highest level of TRA in endemic sera, partly due to its high stringency and reliance on unnaturally high gametocyte densities and on no local malaria vector for African settings, *Anopheles stephensi* [47]. It was recently shown that functional TRA that affects the transmission from natural infections may be missed by the SMFA [48]. The SMFA may thus not be as sensitive to detect natural transmission reducing activity of field serums that were induced against concurrent lower or submicroscopic gametocyte densities. In the current study, we use direct membrane feeding (DMFA) measurements that assess the infectiousness of naturally acquired gametocytes to locally relevant vectors. DMFA experiments were performed during natural infections in all age groups from the entire range of infectious gametocyte densities and allow for the first time a direct assessment of the impact of TRA on natural malaria transmission [25, 41, 49, 50].

Despite the unique richness of the present data, which include hundreds of concurrent measurements of parasite densities, serological markers, and human infectiousness, the underlying dynamics between longitudinal sampling points are complicated: seasonality impacts the timing of new infection events; age-dependent blood-stage immunity modulates the distribution of oscillating parasite densities; and recent gametocyte exposure drives changes in sexual-stage antibody concentrations. In order to assist in the interpretation of statistical relationships observed in these data, we have taken an existing model of infection and immunity dynamics in Burkina Faso [51] and extended it to include boost and decay of sexual-stage antibodies. The goal of the present modeling work is not to identify the optimal model structure or parameterization for gametocyte density and sexual-stage antibody dynamics; that will require significantly more temporal resolution in future longitudinal surveys. Rather, the goal throughout will be to compare which features of the data might follow directly from a set of plausible assumptions about unobserved dynamics, and to highlight how this should impact their interpretation.

## Methods

### Ethics statement

The study received ethical clearance from the Ethical Review Committee of the Ministry of Health of Burkina Faso (MS/MESSRS/N° 2007–035). Study procedures, risks and benefits were explained to participants and written informed consent obtained from adults and parents/guardians of children prior to enrolment. Negative and positive control plasma samples [25] used in the ELISA were anonymized and provided by Sanquin blood bank (Nijmegen, the Netherlands) to the Department of Medical Microbiology, Radboud University Medical Center, Nijmegen, for malaria research purpose and approved by institutional Review Board for use in the present study.

### Field activities

The serological and oocyst data were collected through a longitudinal study on the infectious reservoir [11]. Data are deposited in the Dryad repository: (http://dx.doi.org/10.5061/dryad.v60jk42) [52].

**Fig 1:**
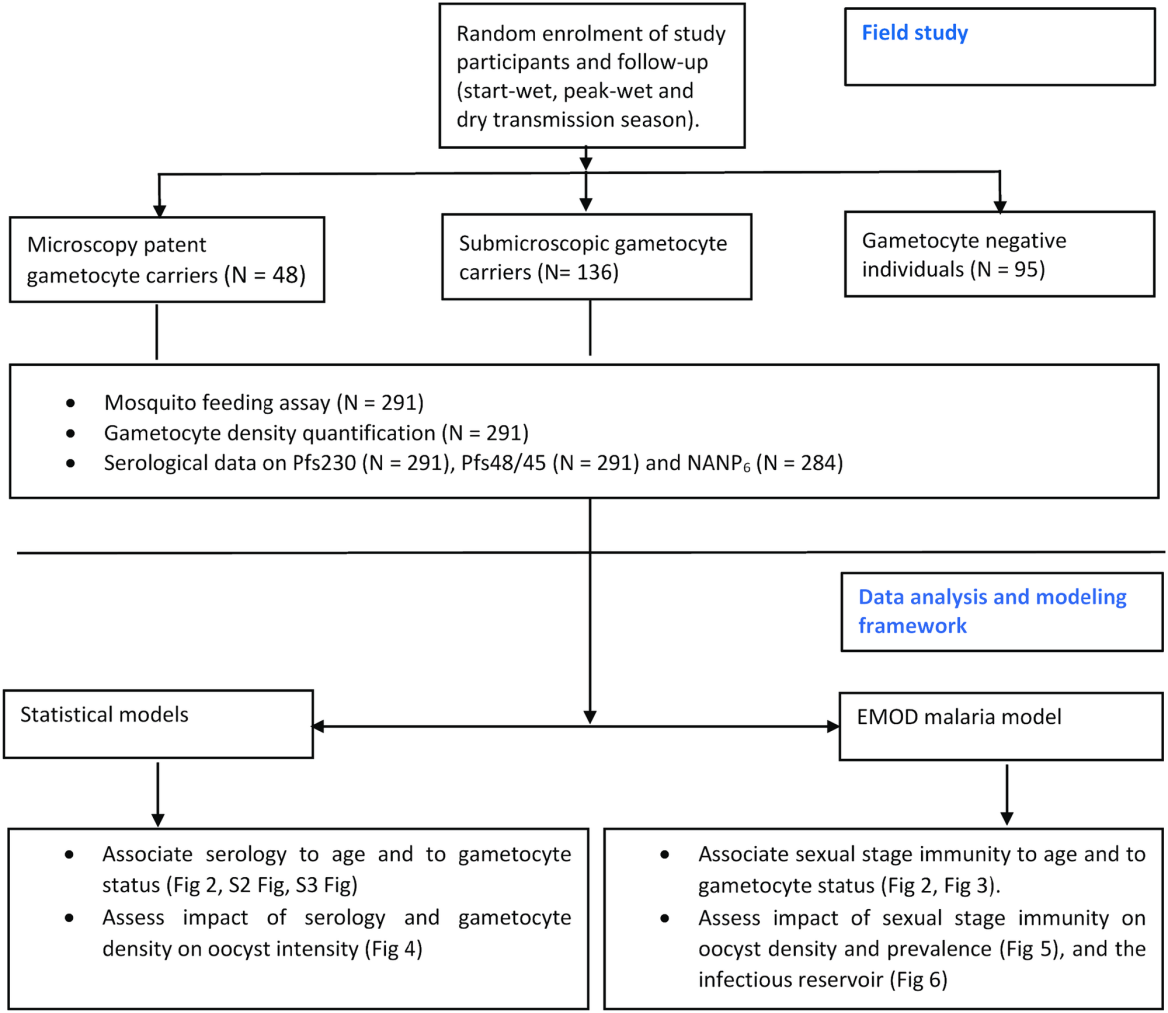
Field study design, data analysis and modeling framework. N = number of observations.

The study design and procedures for data analysis and modeling are summarized in Fig 1; clarifying where conventional statistical models were used to present and analyze study data and where data were incorporated into the EMOD malaria model to simulate their impact on malaria transmission and the human infectious reservoir for malaria. Study participants of all ages living in a hyper-endemic and seasonal malaria transmission setting of Burkina Faso were randomly recruited from four age groups (< 5 years, 5–14 years, 15–30 years and above 30 years of age) in proportions that reflect local demographic proportions (20%, 30%, 25% and 25% respectively). Individuals were invited to participate; and the first to arrive were enrolled until samples sizes for the different age groups were reached.

To study infectivity to mosquitoes in relation to parasite density, age, season and sexual stage immunity, participants were invited to donate blood samples. These samples were used for parasite detection, mosquito feeding assays and assessment of specific anti-gametocyte antibodies (prevalence and ELISA optical density as proxy for antibody density). These assessments were performed at the start of the wet season, the peak of the wet season and during the following dry season.

#### Parasite detection

To improve our understanding of the relationships among human-to-mosquito transmission, gametocyte density and sexual stage immunity, *P*. *falciparum* asexual parasite and gametocyte densities were microscopically determined from blood slides by screening 500–2000 white blood cells (WBC) while assuming a standard count of 8000 WBC/μL of blood suggesting a detection limit of 16 parasites/μL for trophozoites and 4–16 gametocytes/μL for gametocytes. Nucleic acids were extracted from 100 μL aliquots of venous blood samples using the guanidium isothiocyanate (GuSCN) / silica procedure [53] for the quantification of mature gametocytes by Pfs25 mRNA quantitative nucleic acid sequence based amplification (QT-NASBA) [54] with a detection limit ≥ 0.01 gametocyte/μL.

#### Mosquito feeding experiments

Direct membrane feeding assays (DMFA) were performed to assess human-to-mosquito transmission capability [20]. A venous blood sample was offered to approximately fifty 4–5-day-old female *An*. *gambiae* mosquitoes via an artificial membrane attached to a water-jacketed glass feeder maintained at 37°C. At the end of the feeding experiment, fully blood fed mosquitoes were kept and fed with glucose at 29°C for 7 days to allow the development of mature oocysts on the mosquito gut. On day 8, dissected mosquito guts (Mean number = 40) were stained with 1% mercurochrome and screened for oocysts using light microscopy. The numbers of oocysts were confirmed by two independent microscope readers, counted and recorded per mosquito.

#### Serological assays

To study the effect of naturally acquired immunity on human-mosquito transmission, plasma from the same blood samples used for parasite detection and mosquito feeding assays were screened for *P*. *falciparum* sexual stage immunity against Pfs48/45 and Pfs230 antigens. Anti-sporozoite immunity against NANP_6_ antigen was assessed and used as a marker of exposure to sporozoites as well as a non-transmission reducing (human-to-mosquito) immune marker to improve interpretation of Pfs48/45 and Pfs230 anti-gametocyte immune profiles. Antibody titers were not determined and sera were tested at one dilution per antigen. The optical density (OD) at this dilution was used as a proxy for antibody density, as commonly done.

#### Pfs48/45 and Pfs230 ELISA

Mature *P*. *falciparum* NF54 strain gametocytes produced in an automated static culture system were purified [55] and used to extract Pfs48/45 and Pfs230 native antigens. Briefly, the presence of anti-Pfs48/45 and anti-Pfs230 immunoglobulin G (IgG) antibodies in participant’s plasma samples were determined by coating 10μg/mL of anti-Pfs48/45 rat monoclonal antibody 85RF45.3 and anti-Pfs230 rat monoclonal antibody 63F6D7-F(ab). Free sites were blocked with milk (Marvel, Premier International Foods Ltd, Spalding, Lincs, United Kingdom). Pfs48/45 and Pfs230 antigens from gametocyte extract (250,000 gametocytes equivalents/well) were captured overnight. Plasma samples at 1:100 dilution were added to the wells prepared with and without antigen (control wells) for 2 hours incubation. The plate was then washed and specific bound IgG antibodies were detected by reaction to Goat-anti Human IgG-Peroxydase (H+L; Pierce). Wells were further washed and subsequently incubated with tetramethyl benzidine (TMB) substrate. The color reaction was stopped with 4N H2SO4, and the optical density (OD) was read at 450 nm in an Anthos 2001 Microplate Reader (Labtec BV). All plasma samples were tested in duplicate. Three non-immune plasma samples randomly selected from Dutch blood bank donor’s plasma samples were used as negative controls and one positive-control plasma of a Dutch expatriate who had been exposed to malaria for almost 30 years in Sub-Saharan Africa were included per plate. The cutoff was calculated as the mean OD of negative controls plus three standard deviations. A sample was considered positive if its OD value density was above the cutoff.

**NANP**_**6**_ **ELISA** consisted of coating 1μg/mL of NANP_6_ antigen in flat bottom high binding microdensity 96 wells plate (NUNCTM Maxisorp, Nalge Nunc International Corp, Life Techn, The Netherlands). After overnight incubation at 4°C, coated plates were washed and free sites blocked with milk as above. Subsequently, the blocking buffer was washed off and plates incubated at room temperature with 1:100 diluted plasma samples. Plates were then washed and incubated with rabbit anti-human IgG-Peroxidase (Dako, P-214) before reaction with the substrate. The rest of the procedure corresponds to that describe for Pfs48/45 and Pfs230 ELISA. A plasma sample was considered positive if the OD value was greater than three standard deviations above the mean of the negative control plasma samples [24]. All ELISA experiments were well standardized and reproducible. Outliers in OD densities were kept in the data analysis as common natural events due to individual variations upon exposure to intense malaria transmission and or other co-infections.

### Sample size

As described elsewhere [11], we were not able to estimate samples sizes based on experimental mosquito’s infection rates due to the paucity of age and season-related mosquito feeding data. Serological sampling for the current study was never presented but part of a previous study that compared parasite prevalence between age groups. For that study question, that informed sample size, pre-existing age profiles of parasite rates determined by QT-NASBA in the present study area [15] were used. An enrollment and follow-up of 50 volunteers < 15 years and 50 volunteers ≥ 15 years of age would allow over 85% power to detect a significant difference in gametocyte prevalence between the two age groups at the two-tailed 5% significance level.

### Data analysis using statistical models

All statistical analyses were performed in R version 3.3.1. Gametocyte positive samples in the Pfs25 mRNA QT-NASBA were considered submicroscopic if negative by microscopy. Median densities (optical density) of Pfs230 and Pfs48/45 antibodies were categorized by age group (1–4, 5–14 or 15+ years), season (start-wet, peak-wet or dry) and gametocyte density (sub-microscopic or microscopic) to describe antibody density dynamics. The proportion of seropositive individuals and infected mosquitoes were categorized by age group (1–4, 5–14, 15–30 and 30+ years) to describe antibody prevalence and mosquito infection dynamics. A Wilcoxon rank sum test was used to compare the mean oocyst density between samples seronegative versus seropositive to Pfs48/45 and Pfs230 antigens at different gametocyte densities (Submicroscopic, 0.01–16, 17–100, ≥100 gametocytes/μL). A statistical test was considered significant if p ≤ 0.05.

A generalized linear mixed effect model (GLMM) was used to assess the impact of *P*. *falciparum* sexual stage immunity on oocyst intensity. The intensity of *P*. *falciparum* oocysts per individual mosquito midgut and per participant was used in this model as a proxy of human-to-mosquito transmission capability. Because natural malaria infections in endemic areas are unique to individuals, sophisticated regression analyses that account for confounding factors are required to isolate the effect of specific immune factors on malaria transmission. The GLMM grounded in R software AD Model Builder framework accounting for zero inflation, over-dispersion and repeated measurements was used to estimate the effects of anti-Pfs48/45 and Pfs230 antibodies on oocyst intensity assuming a negative binomial distribution of oocysts counts in the mosquito population. Only gametocyte positive individuals (i.e. with density ≥ 0.01 gametocyte/μL by QT-NASBA) were included in the model. Factors influencing human-to-mosquito transmission including participant’s age, season and microscopy asexual parasite prevalence were included as covariates in the model in addition to gametocyte density as key predictor. Given that all mosquitoes were exposed to a constant duration of blood meal uptake in a repeated measurement scenario, subject’s identity and number of dissected mosquitoes were included as random effect and offset variables respectively to allow for within-subject correlation and normalization of the variable number of dissected mosquitoes across individuals. Transmission reducing-effects of model were estimated based on corresponding adjusted regression coefficients. Regression coefficients (β values) that associated with Pfs48/45 and Pfs230 antibodies were transformed into odd ratios (*e*^*β*^) and the transmission-reducing activity of *P*. *falciparum* sexual stage immunity on oocyst intensity defined as *TRA*_*oocyst*_ = 1 − *e*^*β*^.

### Modeling the dynamics of sexual-stage immunity and the infectious reservoir for malaria

A birth cohort of 100 individuals was simulated over a period of 50 years using the EMOD model configured as in [51] with incidence of new infections matching the seasonal transmission characteristics of Burkina Faso. Simulated sexual-stage antibody concentrations for Pfs48/45 and Pfs230 were boosted in response to simulated gametocyte densities each day before decaying with a 1-month half-life—consistent with the immunoglobulin G half-life [56, 57] and with repeat observations from this and other cohort studies (S1 Fig). Procedures for antibody half-life estimates are described in S1 Appendix in section 1 and 2. An alternative approach to estimate antibody half-life that fits a simple equation to time between serological measurements estimated half-lives of 46.4 days for Pfs230 and 142 days for Pfs48/45 (see S1 Appendix – Section 1). The reason for the disparity in estimates for Pfs48/45 is uncertain but may be related to antibody boosting by regular antigen re-exposure that is evident in the study site [11, 58, 59] and not incorporated in the simple antibody decay model. Boosting rates were fitted for each antigen to match the observed features of antibody density distributions—median densities and inter-quartile ranges by age, season, and current gametocyte density—under the structural assumptions that higher gametocyte densities may boost more strongly and that age-dependent cumulative exposure may impact the magnitude of the boost and the inter-individual variation within a smoothness constraint (S1 Appendix). An age-matched set of simulated trajectories was sampled at the 3 time points corresponding to the survey data.

## Results

One hundred and twenty-eight study participants with both *P*. *falciparum* sexual stage immunity and mosquito’s feeding data were included in the data analysis. On the three occasions of seasonal sampling time points, 66 of the study participants were visited on three occasions, 31 twice and 31 once resulting in a total of 291 blood samples. NANP_6_ antibody density and parasite density by QT-NASBA were obtained from 284 and 285 respectively of the 291 blood samples.

### Pfs48/45, Pfs230 and NANP_6_ antibody profiles

The overall prevalence of NANP_6_ antibodies was 78.1% (222/284) and this antibody prevalence increased significantly with age (Adjusted β (regression coefficient) = 0.838, se = 0.288, p = 0.0037, Table 1). The density of NANP_6_ antibodies also increased with age (β = 1.032, se = 0.29, p < 0.001) and season with the lowest levels recorded at the start (0.4752) of the wet season compared to the peak (0.7165) and dry season (0.8302) (β = -0.387, se = 0.164, p = 0.018).

**Table 1:** Pfs48/45 and Pfs230 antibody prevalence and densities from Field data and model simulations.

Antigen	Pfs48/45		Pfs230		NANP_6_
Data	Field data % (No/Total)	Model % (No/Total)	Field data % (No/Total)	Model % (No/Total)	Field data % (No/Total)
Age group (years)					
<5	13.51 (5/37)	14.0 (210/1500)	16.21 (6/37)	17.4 (261/1500)	38.9 (14/36)
5–14	26.80 (26/97)	24.23 (727/3000)	24.74 (24/97)	26.13 (784/3000)	64.13 (59/92)
15–29	27.17 (25/92)	25.46 (1146/4500)	41.30 (38/92)	40.0 (1800/4500)	93.41 (85/91)
30+	15.38 (10/65)	18.08 (1085/6000)	46.15 (30/65)	48.78 (2927/6000)	98.46 (64/65)
Total	22.68 (66/291)	21.12 (3868/15000)	33.67 (98/291)	38.48 (5772/15000)	78.17 (222/284)
Antibody density					
Mean	0.6542	0.6187	0.7673	0.7653	
Standard deviation	0.3681	0.3662	0.5018	0.5262	
Min	0.202	0.0151	0.052	0.0194	
Max	2.851	2.0320	2.4653	2.4926	
Gametocyte % Prevalence (n/N)	66% (188/285)	69% (10397/15000)	66% (188/285)	69% (10397/15000)	

**Fig 2:**
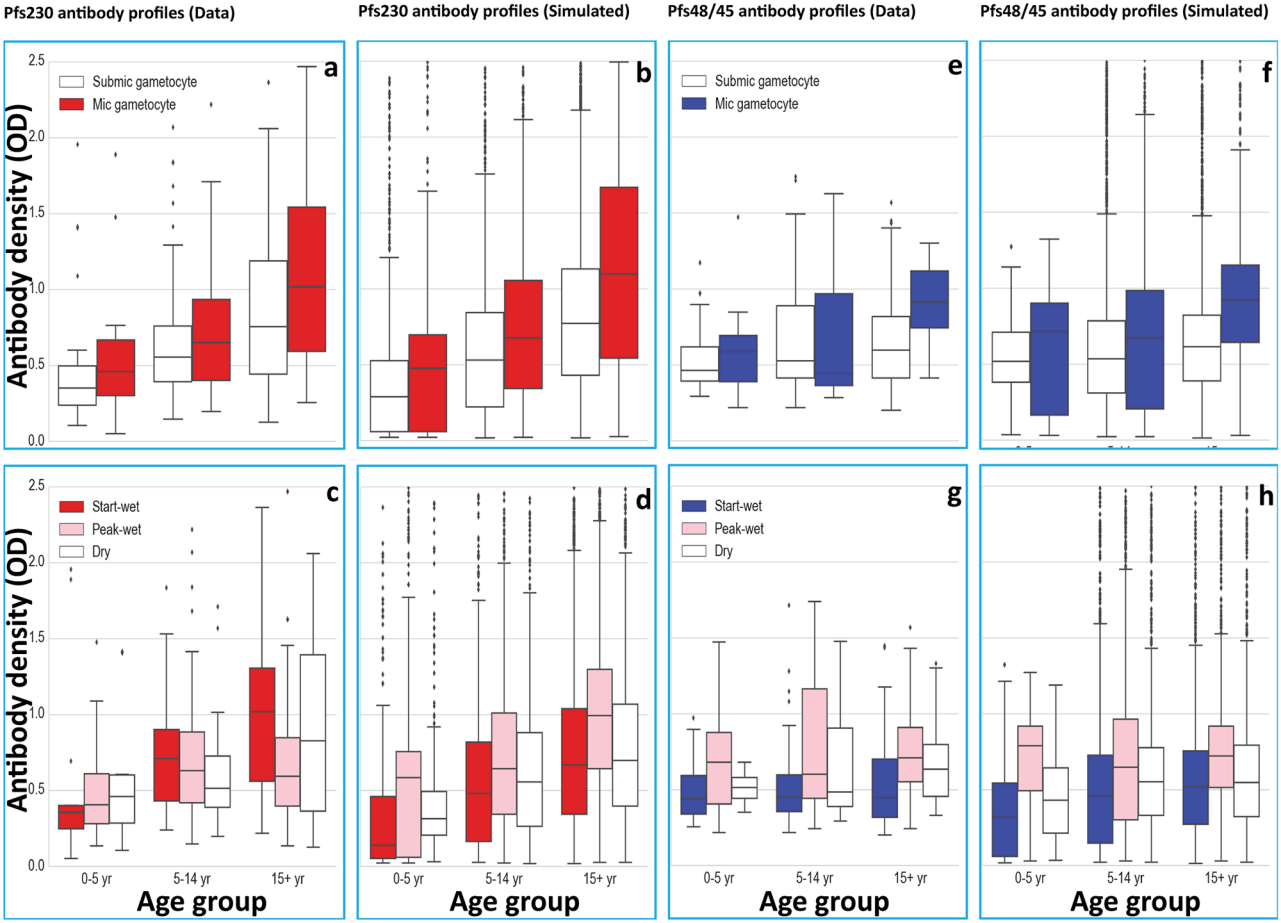
Dynamics of Pfs230 and Pfs48/45 antibody densities by age, season and gametocyte density. Submic gametocyte = Submicroscopic gametocyte densities ranging from 0.01–4 gametocytes/ μL blood thus negative by microscopy and positive by QT-NASBA. Mic gametocyte = gametocyte positive by both microscopy and QT-NASBA. Antibody densities of field study participants were measured in subsamples of n = 37, n = 97 and n = 157 samples in age groups 1–4, 5–14 and ≥15 years of age respectively and shown in panel a for Pfs230 and panel e for Pfs45/45. Antibody densities of simulated individuals were measured in subsamples of n = 1500, n = 3000 and n = 10500 samples in age groups 1–4, 5–14 and ≥15 years of age respectively and shown in panel b for Pfs230 and panel f for Pfs48/45. Antibody densities of field study participants were measured in subsamples of n = 102, n = 96 and n = 93 samples during the start, peak and wet seasons respectively and shown in panel c for Pfs230 and panel g for Pfs45/45. Antibody densities of simulated individuals were measured in subsamples of n = 5000, n = 5000 and n = 5000 samples during the start, peak and wet seasons respectively and shown in panel d for Pfs230 and panel h for Pfs48/45. Error bars show horizontal lines from top indicating maximum antibody density, 75% percentile, median (50% percentile), 25% percentile and minimum antibody density. Dots outside shaded boxes represent outliers in the distribution of antibody densities within the given subsample.

Compared to NANP_6_, a smaller proportion of samples showed reactivity to Pfs48/45 (22.6%) and Pfs230 (33.6%) (Table 1); only 12% (36/291) of the samples reacted to both Pfs230 and Pfs48/45 antigens. Antibody density and seroprevalence were lower for Pfs48/45 compared to Pfs230. Pfs230 antibody prevalence was significantly higher in adults (β = 1.150, se = 0.445, p = 0.0097, Table 1), and adults also had higher relative antibody densities (β = 0.5289, se = 0.2502, p = 0.0345). In contrast to Pfs48/45 (Fig 2g), the prevalence and density of Pfs230 antibodies in individuals ≥15 years of age dropped at the peak of the wet season, although not significantly (Fig 2c).

The prevalence of Pfs48/45 antibody increased with age up to 30 years but declined in older age (adjusted β = 0.871, se = 0.507, p = 0.086, Table 1), and prevalence was higher (adjusted β = 0.470, se = 0.3012, p = 0.12) at the peak of the wet season (31%, 30/96) compared either to the start of the wet season (17%, 18/102) or to the dry season (19%, 18/93). Interestingly, although not statistically significant, the decline in Pfs48/45 antibody after 30 years of age coincided with a drop in the mean gametocyte density in that population from onset (80.5 gametocytes / μL) to peak transmission (58.7 gametocytes / μL).

Pfs48/45 antibody density also showed an age-related trend (Adjusted β = 0.2581, se = 0.2629, p = 0.451) and reached a peak during the wet season (Fig 2e and 2g) although not significantly (β = 0.1064, se = 0.1752, p = 0.544).

**Fig 3:**
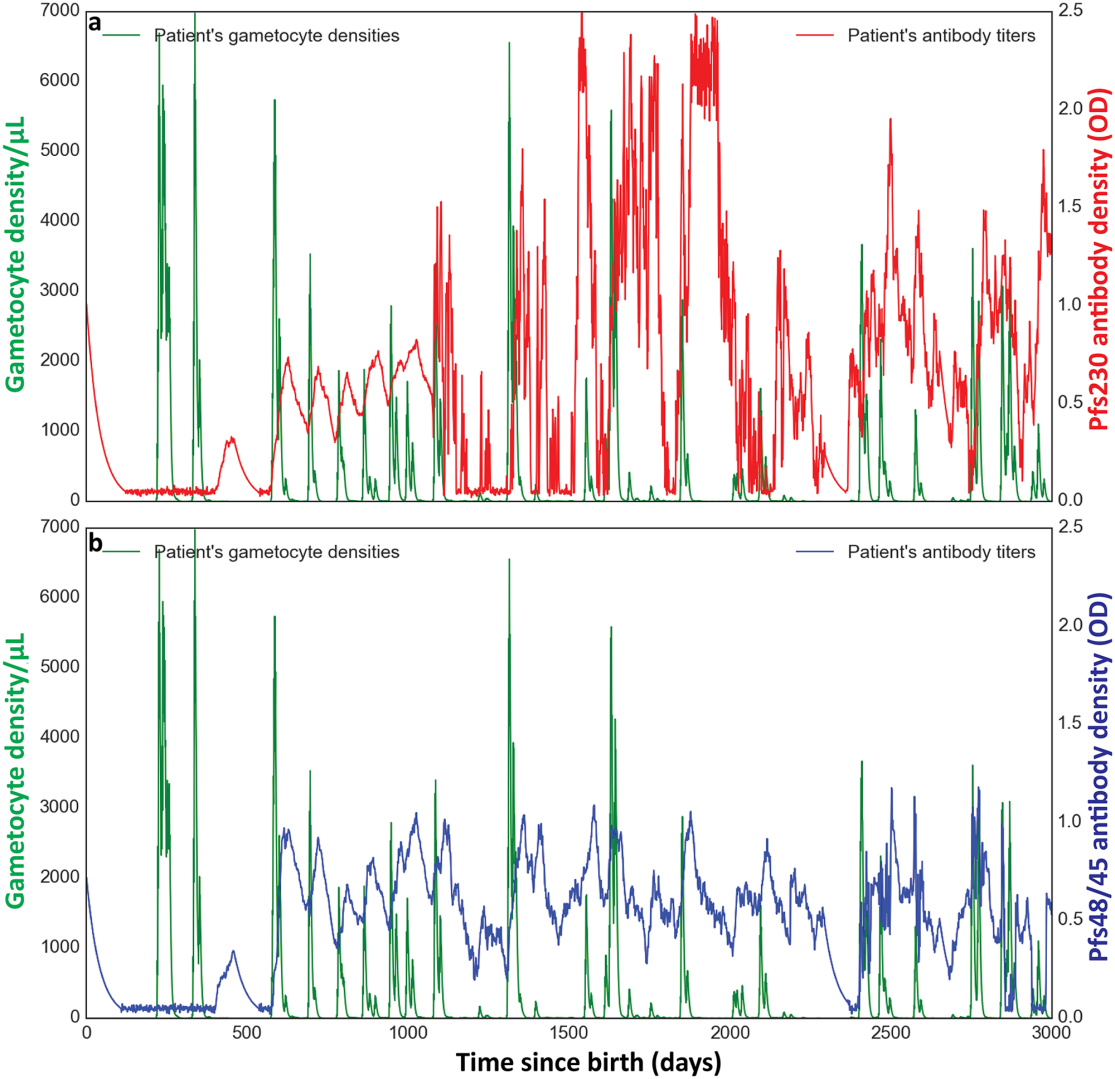
Time series dynamics of Pfs230 (a) and Pfs48/45 (b) antibody response against gametocyte density in a simulated malaria infected individual from birth up to day 3000.

Reflecting on the characteristics of the dynamical model for Pfs230 (Fig 2d) and Pfs48/45 (Fig 2h) that are required to reproduce the age and seasonal trends observed in the surveys, two keys features are apparent. First, a relatively short decay half-life is an important driver of both the seasonal variation and also the inter-individual variation driven by periodic spikes in gametocyte density (Fig 3 shows an example trajectory). Second, a combination of chronic submicroscopic boosting and more efficient boosting with cumulative exposure influences the increasing densities with age, especially for Pfs230.

### *P*. *falciparum* sexual stage immunity associates with concurrent gametocyte density

We further investigated the impact of concurrent gametocyte carriage, either sub-microscopic or microscopic, on measured *P*. *falciparum* sexual stage antibody densities to better understand antibody acquisition and decay upon parasite exposure.

In Fig 2a, under 5, 5–14 and 15+ years old submicroscopic gametocyte carriers presented with 0.3515, 0.6597 and 0.8394 median Pfs230 antibody densities respectively, which were lower though not significantly so, compared to densities in microscopic gametocyte carriers (0.4597, 0.7808, 1.1183 respectively). Similarly, in Fig 2e, under 5, 5–14 and 15+ years old submicroscopic gametocyte carriers presented with 0.4640, 0.525 and 0.594 median Pfs48/45 antibody densities respectively whilst microscopic gametocyte carriers had increased though not significantly so Pfs48/45 median antibody densities (0.5865, 0.4395 and 0.9115 respectively).

GLMM regression models that included age and parasite status showed that microscopic gametocyte carriers were more likely to have higher Pfs230 (Adjusted OR = 1.52, se = 0.302, p = 0.164) and Pfs48/45 antibody densities (Adjusted OR = 1.4, se = 0.3282, p = 0.34) than submicroscopic gametocyte carriers, although not statistically significantly so.

Whilst anti-gametocyte immunity has been hypothesized to be short-lived [39, 60] anti-Pfs230 and anti-Pfs48/45 antibodies were detected in a considerable fraction of individuals without concurrent gametocytes, plausibly reflecting recent gametocyte exposure. Under 5, 5–14 and 15+ years old gametocyte-negative individuals presented with 0.2031, 0.7868 and 0.7936 median Pfs230 antibody densities and 0.5662, 0.6332 and 0.6524 median Pfs48/45 antibody densities, respectively, which tend to be lower than densities in submicroscopic and microscopic gametocyte carriers (S2 Fig). There was no association or trend between NANP_6_ antibodies and concurrent gametocyte density (S3 Fig).

The observed pattern of a small but consistent shift in antibody densities for concurrently detected gametocytes is an expected consequence of the dynamical model for Pfs230 (Fig 2b) and Pfs48/45 (Fig 2f). Quantitatively, it depends on the gametocyte density varying on a timescale that is similar to the antibody decay time and the degree to which higher gametocyte densities resulting in more efficient boosting.

### Impact of sexual stage immunity on oocyst intensity in mosquito feeding assays

*P*. *falciparum* gametocytes were detected in 66% of samples (188/284) by QT-NASBA (Table 1) whilst 32% (96/291) of the total population was infectious to mosquitoes (i.e. infected at least one mosquito). *P*. *falciparum* oocyst counts as enumerated in mosquito’s midguts followed a zero-inflated negative binomial distribution (S1 Appendix – Fig 3). Among the 11,440 dissected mosquitoes, 886 mosquitoes were found to be infected with one or more oocysts ranging from 1 to 97 oocysts.

### Oocyst distribution associates with age, season and gametocyte density

Predictors of oocyst intensity were tested in a GLMM, and the estimated coefficients are shown in Table 2. The intensity of oocysts significantly decreased with age and was lower in the dry season as compared to the transmission season. In mosquitoes that fed on blood samples from individuals ≥ 15 years of age, the intensity of oocysts was significantly lower compared to oocyst intensity of mosquitoes fed on blood samples from individuals 5–14 (p = 0.003) and under-5 years old children (p = 0.047, Table 2). Similarly, season was related to oocyst with an increased oocyst intensity at the start (p = 0.075) and peak of the wet season (p < 0.001, Table 2). Carriers of gametocytes (≥100 gametocytes / μl blood) or microscopic asexual parasites positively associated with oocyst intensity (p < 0.001 for both covariates, Table 2).

**Table 2:** Generalized linear mixed model (GLMM)* adjusted effects of Pfs48/45 antibody responses on oocyst intensity.

Factor		Individuals No	Infected mosquitoes No/Total	Oocysts No	Adjusted β (se)	P-value
Age group (years)						
	30+	65	12/2579	13	0.0 (Ref.)	
	15–30	92	142/3546	1351	0.453 (1.171)	0.698
	5–14	97	454/3857	1967	3.258 (1.104)	0.003
	<5	37	278/1458	1806	2.234 (1.126)	0.047
Season						
	Dry	93	120/3532	1388	0.0 (Ref.)	
	Peak wet	96	350/3692	1849	1.060 (0.286)	<0.001
	Start wet	102	416/4216	1900	0.434 (0.244)	0.075
Microscopy asexual parasite^¥^						
	Absence	157	187/5986	473	0.0 (Ref.)	
	presence	119	663/4846	4591	1.072 (0.231)	<0.001
Gametocytes by QT-NASBA, No/ μL^¥^						
	<16	150	98/5738	193	0.0 (Ref.)	
	16–100	52	176/2053	431	0.475 (0.321)	0.139
	≥100	83	604/3367	4500	1.087 (0.320)	<0.001
Pfs48/45 antibody						
	Absence	225	722/8957	4721	0.0 (Ref.)	
	Presence	66	164/2483	416	-0.801 (0.239)	0.0004

ref. = reference, β = regression coefficient, se = standard error,

* = GLMM accounting for zero inflated negative binomial distribution, random effects and within-individual correlation for repeated measurements.

^¥^These groups presented with different sample sizes of participants (n = 277 for Microscopy asexual parasite and n = 285 for Gametocytes by QT-NASBA) and therefore exhibit smaller sample sizes of dissected mosquitoes.

### Oocyst intensity associates with Pfs48/45, Pfs230 but not NANP_6_ antibodies

By investigating the interactions between oocyst counts and antibody densities, adjusting for gametocyte density, we were able to estimate the transmission reducing activity (TRA) of specific antibody densities.

**Fig 4:**
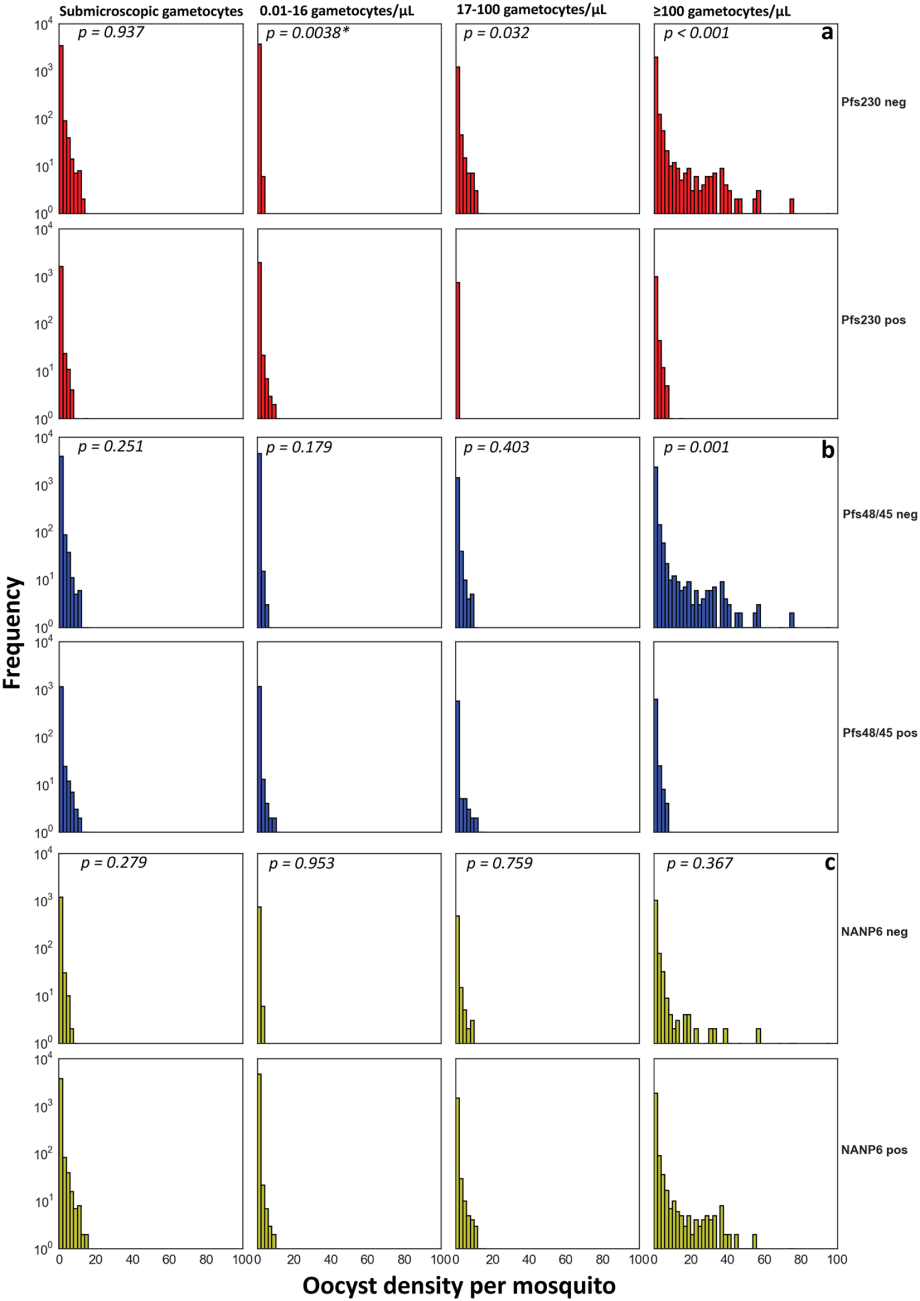
Distribution of oocyst intensity in the presence and absence of Pfs230 (a), Pfs48/45 (b) or NANP6 (c) antibody at different gametocyte densities. Antibody densities of samples seronegative for Pfs230, Pfs48/45 and NANP_6_ ranged 0.202–0.8784, 0.052–0.8784 and 0.0465–0.293 respectively. Antibody densities of samples seropositive for Pfs230, Pfs48/45 and NANP_6_ ranged 0.8784–2.851, 0.8784–2.4653 and 0.293–3.5 respectively. Number of participants with submicroscopic, 0.01–16, 17–100 and ≥ 100 gametocytes/μL were 136, 53, 52 and 83 respectively. Number of mosquitoes fed on submicroscopic, 0.01–16, 17–100 and ≥ 100 gametocytes/μL were 3633, 1314, 1306, 2311 for Pfs230 seronegative samples and 1671, 709, 747, 1056 for seropositive samples respectively. For Pfs48/45 seronegative samples, the numbers were 4107, 1500, 1460, 2702 and 1197, 523, 593, 665 for seropositive samples respectively. For NANP6 the numbers were 1216, 464, 451, 1208 for seronegative samples and 4021, 1559, 1572, 2122 for seropositive samples. *Note that significance indicates that samples positive for Pfs230 antibodies present with higher oocyst intensity.

Fig 4 illustrates TRA in relation to Pfs230, Pfs48/45 and NANP_6_ antibodies at various gametocyte densities determined by QT-NASBA. At gametocyte density ≥100 gametocytes/μL, individuals seropositive to both Pfs230 and Pfs48/45 associated with an order of magnitude reduced oocyst intensities (11 and 11.3-fold respectively, Fig 4a and 4b) in comparison to seronegative individuals (*p* < 0.001). At gametocyte density of 17–100 gametocytes/μL, samples seropositive for Pfs230 significantly reduced oocyst intensity compared to samples seronegative for Pfs230 (*p* = 0.03, Fig 4). The same was not true for samples seropositive for Pfs48/45 (Fig 4). At gametocyte density below 16 gametocytes/μL or at the submicroscopic level, we observed no statistically significant impact for Pfs230 or for Pfs48/45 seropositivity on oocyst intensity (*p* = 0.937 and *p* = 0.251 respectively).

The GLMM regression analysis estimated—after adjustment for age, season, gametocyte density and microscopically detectable asexual parasites—a 55.1% and 70% reduction in oocyst intensity respectively for Pfs48/45 antibody (p < 0.001, Table 2) and Pfs230 (p < 0.001, Table 3). No reduction of oocyst intensity associated with NANP6 antibodies was observed (Fig 4c).

**Table 3:** Generalized linear mixed model (GLMM)* adjusted effects of Pfs230 antibody responses on oocyst intensity.

Factor		Individuals No	Infected mosquitoes No/Total	Oocysts No	Adjusted β (se)	P-value
Age group (years)						
	30+	65	12/2579	13	0.0 (Ref.)	
	15–30	92	142/3546	1351	0.381 (1.279)	0.7
	5–14	97	454/3857	1967	2.106 (1.161)	0.06
	<5	37	278/1458	1806	3.196 (1.209)	0.008
Season						
	Dry	93	120/3532	1388	0.0 (Ref.)	
	Peak wet	96	350/3692	1849	0.007 (0.233)	0.9
	Start wet	102	416/4216	1900	-0.081 (70.206)	0.6
Microscopy asexual parasite						
	Absence	157	187/5986	473	0.0 (Ref.)	
	presence	119	663/4846	4591	1.631 (0.232)	<0.001
Gametocytes by QT-NASBA, No/ μL						
	<16	150	98/5738	193	0.0 (Ref.)	
	16–100	52	176/2053	431	2.048 (0.312)	<0.001
	≥100	83	604/3367	4500	2.797 (0.311)	<0.001
Pfs230 antibody						
	Absence	193	694/7633	4710	0.0 (Ref.)	
	Presence	98	192/3807	427	-1.19 (0.243)	<0.001

ref. = reference, β = regression coefficient, se = standard error,

* = GLMM accounting for zero inflated negative binomial distribution, random effects and within-individual correlation for repeated measurements. ^¥^These groups presented with different sample sizes of participants (n = 277 for Microscopy asexual parasite and n = 285 for Gametocytes by QT-NASBA) and therefore exhibit smaller sample sizes of dissected mosquitoes.

**Fig 5:**
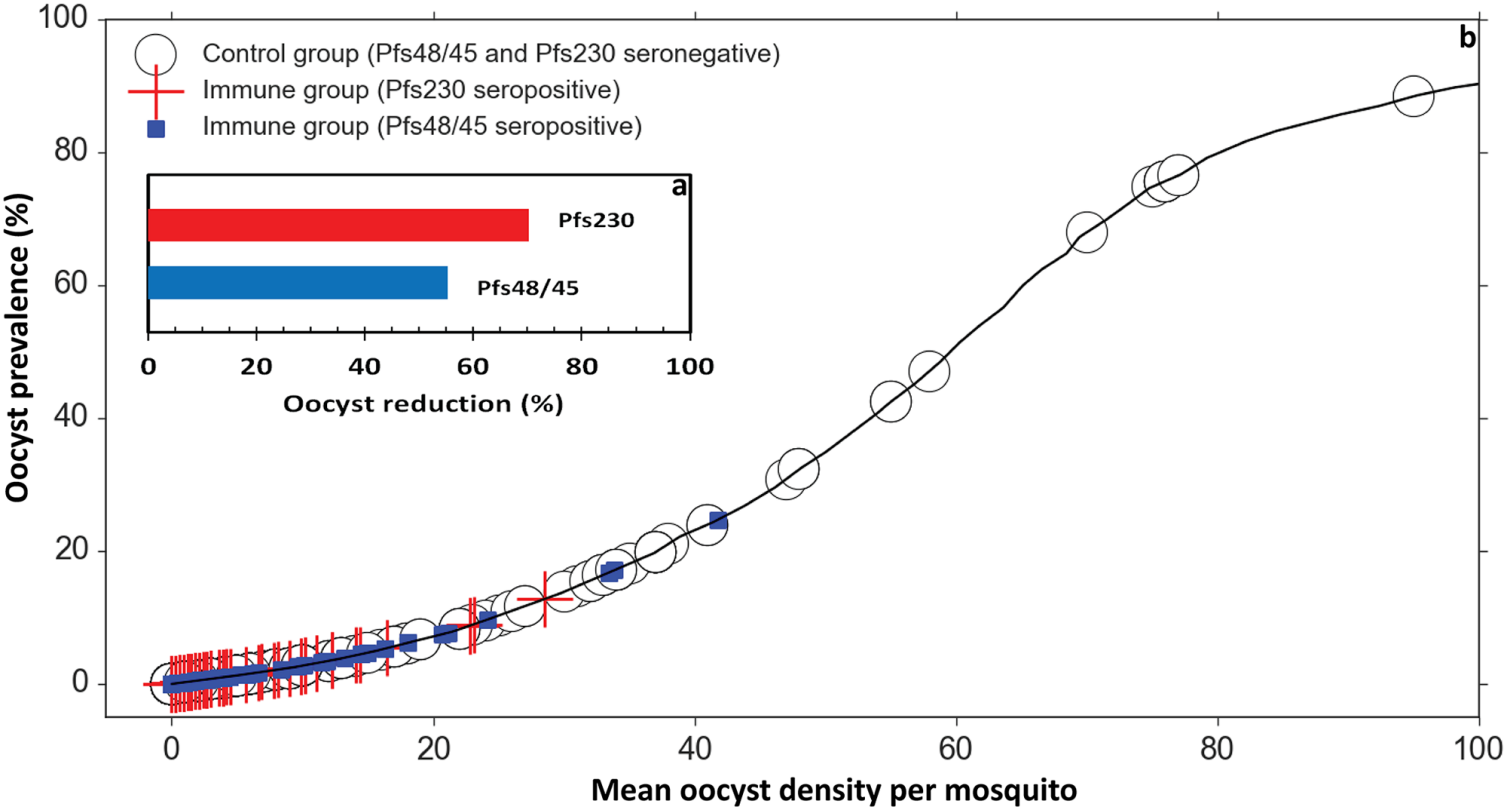
Predicted prevalence of infected mosquitoes in individuals seronegative for Pfs230 and Pfs48/45 (control), individuals seropositive for Pfs230, Pfs48/45 or either antigen. Procedures of simulation of oocyst intensity and inference of oocyst prevalence data per individual are described in S1 Appendix. Number of observations were 15000 in the control group (all seronegative to either antigen), 5662 (37.7% seropositive) in the group seropositive to Pfs230, 3098 (20.6% seropositive) in the group seropositive to Pfs48/45 and 1804 (12% seropositive) in the group seropositive to either antigen.

Simulated gametocyte densities generated an overall fraction of 39.9% (5998/15000) infectious individuals with an overall mean mosquito infection rate of 5.6% (850/15000) and oocyst intensity ranging from 0 to 94 (including non-infectious individuals). Based on the values of TRA estimated from the GLMM regression analysis, the presence of antibody with specificity for Pfs48/45 or Pfs230 resulted in reductions of 26%, 37% respectively and for both Pfs48/45 and Pfs230 a reduction of 44% in mosquito infection rates (Fig 5).

### Modelling the impact of transmission reducing immunity on the composition and dynamics of the human infectious reservoir

Our findings of an age and season-dependent prevalence and density of antibodies to Pfs48/45 and Pfs230 and a considerable impact of these antibodies on the likelihood of mosquito infection indicate that incorporating sexual stage immunity in transmission models may improve our ability to quantify the contribution of different populations to the infectious reservoir for malaria.

**Fig 6:**
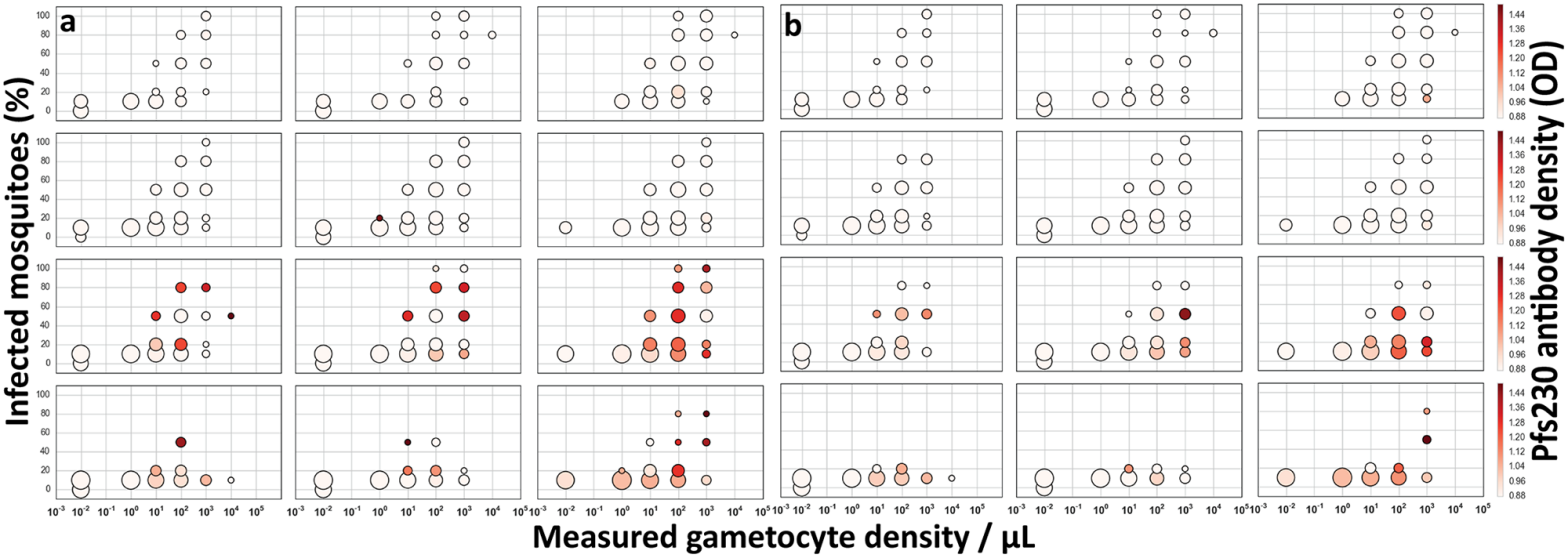
Simulated effect of TRA (Pfs230 TRA alone, Pfs48/45 TRA alone or either) on the association between gametocyte density and mosquito infection prevalence for different age groups and time points in the transmission season. In panel a, the predicted infectious reservoir accounts for measurement uncertainty of true gametocyte density in the absence of sexual stage immunity effect, in panel b, the predicted infectious reservoir accounts for measurement uncertainty of true gametocyte density and for transmission reducing immunity. Simulated mosquito infection rates were estimated using 1500, 3000, 4500 and 6000 mosquito’s feeding outcomes from age groups 1–4, 5–14 15–29 and ≥30 years of age respectively during each season.

Fig 6a incorporates the smearing of true simulated gametocyte densities to account for uncertainty in QT-NASBA derived gametocyte density estimates (S1 Appendix – Fig 5 [61]). This results in a clear over-dispersion of infected mosquitoes spanning a wider range of measured gametocyte density (10^0^–10^4^ gametocytes/μL). But another important consequence is that high gametocyte density measurements will appear to be less infectious in groups with lower true average density (e.g. older ages), since it is proportionately more likely they result from lower true densities in the tails of the measurement uncertainty distribution [62].

Fig 6b additionally includes the effect of TRA from both Pfs230 and Pfs48/45; the color of the bubbles corresponds to the Pfs230 antibody density distribution (as an example) across individuals. The age- and season-dependence of high antibody densities is reflected in the changing shape of the infectious reservoir between Fig 6a and 6b. In general, there was 1.14 (11.3%/9.9%), 1.24 (7.6%/6.1%), 1.45 (3.2%/2.2%) and 1.5-fold (0.6/0.4) decrease corresponding to 12.2, 19.3, 31 and 33% reductions in mosquito infection rate in the <5, 5–14, 15–30, and 30+ year old individuals because of TRA impact, providing an improved fit to the study site data as described elsewhere [11]. There was no seasonal impact of TRA on the proportion of infected mosquitoes.

The shape of the association between oocyst prevalence and oocyst intensity (S1 Appendix – Fig 4 [62]) was not altered by the presence of antibodies against Pfs230 or Pfs48/45 (Fig 5). Both oocyst prevalence and oocyst intensity were lower for experiments on samples with detectable antibodies to these antigens with 70% and 55% as best estimate of the reductions in oocyst intensity related to the presence of antibodies against Pfs230 and Pfs48/45, respectively (Fig 5).

## Discussion

Using a unique set of longitudinal data on malaria-specific antibody responses and infectivity of gametocytaemic individuals to mosquitoes, we showed that *P*. *falciparum* sexual stage immunity significantly reduces the transmission of microscopic but not submicroscopic gametocyte infections. A notable age-dependent decrease in mosquito infection rate is attributed to both Pfs48/45 and Pfs230 immunity in a computational model. Our simulations reflect and further support our field findings that *P*. *falciparum* sexual stage immunity associates with recent exposure to gametocyte density and duration of exposure.

Whilst the presence of antibodies against *P*. *falciparum* sexual stage antigens in endemic populations is well established [24, 39, 49], it is currently unknown how this sexual stage immunity develops in relation to either microscopic or submicroscopic gametocyte carriage and how it subsequently impacts the likelihood of mosquitoes becoming infected by these malaria-infected human hosts. Although there is conflicting evidence on the impact of age and cumulative malaria exposure on sexual stage immunity [63], sexual stage immunity may influence the composition and dynamics of the human infectious reservoir. We followed an all-age cohort of individuals with variable gametocyte exposure over distinct transmission seasons and directly determined their infectiousness to mosquitoes in relation to the presence or density of sexual stage antibodies against gametocyte/gamete antigens Pfs48/45 and Pfs230.

Pfs48/45 is a pre-fertilization protein on the surface of gametocytes and gametes that has a central role in male gamete fertility [45] while Pfs230 antibodies mediate lysis of gametes in a complement-dependent manner [42]. Both antigens are transmission blocking vaccine candidates that elicit up to 100% functional transmission reducing immunity in animal models [45, 64]. In the present study, antibodies against Pfs48/45 and Pfs230 were more commonly observed in samples with high concurrent gametocyte densities [39, 65]. We did not directly assess antibody titer by diluting participant plasma samples but used the optical density in the ELISA assays as indicator of antibody density, as commonly done [66]. Our simulations indicate a short half-life of anti-Pfs48/45 and anti-Pfs230 antibodies [39]. Although long-lived antibody responses to gametocyte antigens have been observed in expatriates who retained functional antibodies for several years after returning from malaria endemic settings [25], most of the field evidence supports the hypothesis that gametocyte immune responses are more short-lived than asexual responses [49, 67]. Our estimates of an antibody half-life of approximately 1 month for both anti-Pfs48/45 and anti-Pfs230 antibodies must be interpreted with caution. Although the estimates are in line with that from an independent cohort study from a similar setting in Burkina Faso (see S1 Fig), an alternative, simpler approach to estimating antibody half-life that assumes an antibody decay as function of time rather than accounting for parasite exposure in this time window estimated a longer half-life of anti-Pfs48/45 antibodies. In addition, our ELISA methodology that utilizes native gametocyte antigen may be limited in sensitivity and may thus have classified samples with low antibody densities as antibody negative. Because the functionality of sexual stage antibodies is strongly dependent on density [39, 68, 69] we nevertheless consider it unlikely that we have missed functionally relevant antibody densities in the current study. At microscopically detectable gametocyte densities, the presence of anti-Pfs48/45 and anti-Pfs230 antibodies had a pronounced effect on the proportion of mosquitoes that became infected and the oocyst burden in these mosquitoes.

Although antibody densities have been previously associated with TRA in both SMFA [39, 68] and DMFA [69], these studies were all performed in selected high-density gametocyte carriers. Our study is the first to quantify its impact on the infectious reservoir for malaria by concurrent use of membrane feeding assays, antibody assessments and sensitive gametocyte detection. We observed that the presence of antibody with specificity for Pfs48/45, for Pfs230 or for either resulted in a reduction of 26, 37 and 44% in mosquito’s infection rate respectively. We further found that incorporating sexual stage immunity improved the age-related dynamics of the infectious reservoir in simulations. This could be explained by the age-related increase in Pfs48/45 and Pfs230 antibody prevalence and densities. These antibody responses did not explain the comparatively lower mosquito infection rates in the dry season when both antibody prevalence and mosquito infection rates were lower [11]. Older infections surviving the long dry season [70] appear to be less infectious to mosquitoes by a mechanism independent of TRA [71].

We observed a direct relationship between sexual stage antibody responses and recent / concurrent exposure to gametocytes [24, 60, 67, 68]. We thus used gametocyte density and duration of exposure (rather than age as proxy for cumulative exposure), as parameters to the model acquisition of *P*. *falciparum* sexual stage immunity. Our simulations closely replicate our field observations suggesting that age is unlikely to play an independent role in the dynamics of Pfs48/45 and Pfs230 immunity.

However, given the higher Pfs230 antibody density in adults, we could not fully rule out a role of age on the acquisition of sexual stage immunity. Indeed, an age-dependent expansion of B cell memory may be an explanation for the observed patterns, as could be the persistent exposure to gametocyte antigens due to continued low-density gametocytemia, as observed in 40% of adults in the area [11]. While antibody data from three seasonal sampling points allowed us to infer many characteristics of antibody boosting and waning dynamics, there remains significant uncertainty in relation to dependency of antibody acquisition or maintenance in relation to gametocyte density and duration of gametocyte carriage. Future longitudinal studies with more frequent sampling will help refine our understanding in these areas specifically.

To conclude, the present study provides novel insights into how *P*. *falciparum* sexual stage immunity is acquired in seasonal transmission settings and how it impacts infectivity to mosquitoes during natural infections and the human infectious reservoir for malaria. Our finding that *P*. *falciparum* sexual stage immunity has very limited impact on the transmissibility of submicroscopic gametocytes may contribute to previous findings on their important role in sustaining malaria transmission [11, 32, 72]. The finding that *P*. *falciparum* sexual stage immunity associates with concurrent submicroscopic and microscopic gametocyte density has implications for the future deployment of transmission blocking vaccines where antibody boosting densities may be dependent on recent high-density gametocyte exposure. Lastly, we show that *P*. *falciparum* sexual stage immunity significantly reduces the infectious reservoir in an age-dependent manner, legitimizing its incorporation in mathematical models that may be used for projecting the infectious reservoir in the context of malaria elimination and for testing future transmission-blocking interventions *in silico*.

## Supporting information

S1 Fig Individual level of Pfs230 and Pfs48/45 antibody densities in the present and an independent cohort study [24] from Burkina Faso and best model fits of antibody profiles using estimated antibody half-lives. Plasma samples of the independent cohort study were collected in June, August and December of the same year (2002) as described elsewhere [24]. A total of 78 individuals were sampled at least twice (13 were visited 3 times and 65 twice) while 246 were seen once (N = 415 ELISA experiments). Antibody densities were measured using the same ELISA methodology as described in the present study and have never been published. Field plasma antibody densities are shown in panel a and c (the present study) and in panel e and g (independent cohort study). Simulated antibody profiles are shown in panel b and d for half-life of 28 days (the present study) and in panel f and h for half-lives 26 and 32 days (independent cohort study).
(TIF)

S2 Fig Levels of Pfs230 and Pfs48/45 antibody densities per gametocyte carriage status and age of study participants. (TIF)

S3 Fig NANP_6_ antibody density by age and concurrent gametocyte density. Antibody densities of study participants were measured in subsamples of n = 37, n = 97 and n = 157 samples in age groups 1–4, 5–14 and ≥15 years of age respectively. Error bars show horizontal lines from top indicating maximum antibody density, 75% percentile, median (50% percentile), 25% percentile and minimum antibody density. Dots outside shaded boxes represent outliers in the distribution of antibody densities within the given subsample.
(TIF)

S1 Appendix Supplemental methods. (PDF)
