# Supplementary figures and images for: Modeling the impact of Plasmodium falciparum sexual stage immunity on the composition and dynamics of the human infectious reservoir for malaria in natural settings
Source: PLoS Pathog. 2018 May 9;14(5):e1007034. doi: 10.1371/journal.ppat.1007034 (PMC5962096; doi:10.1371/journal.ppat.1007034)

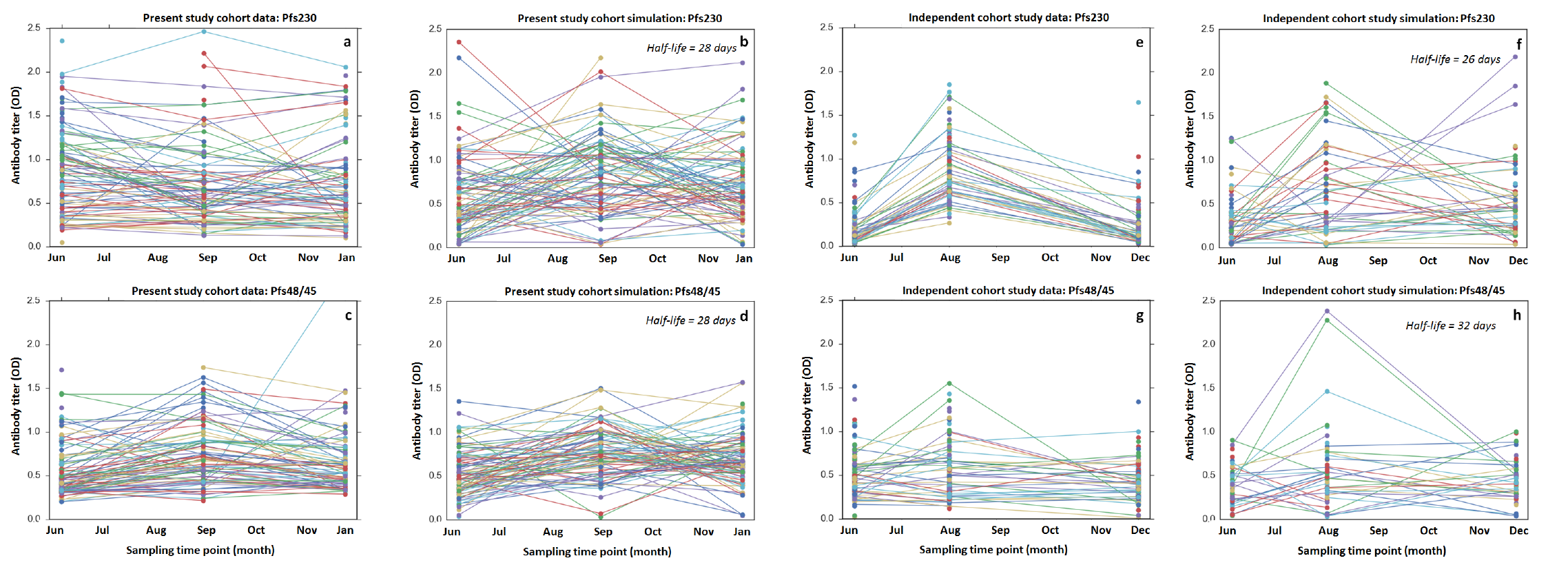

Supplement: S1 Fig — Plasma samples of the independent cohort study were collected in June, August and December of the same year (2002) as described elsewhere [24]. A total of 78 individuals were sampled at least twice (13 were visited 3 times and 65 twice) while 246 were seen once (N = 415 ELISA experiments). Antibody densities were measured using the same ELISA methodology as described in the present study and have never been published. Field plasma antibody densities are shown in panel a and c (the present study) and in panel e and g (independent cohort study). Simulated antibody profiles are shown in panel b and d for half-life of 28 days (the present study) and in panel f and h for half-lives 26 and 32 days (independent cohort study). (TIF) [file ppat.1007034.s001.tif]

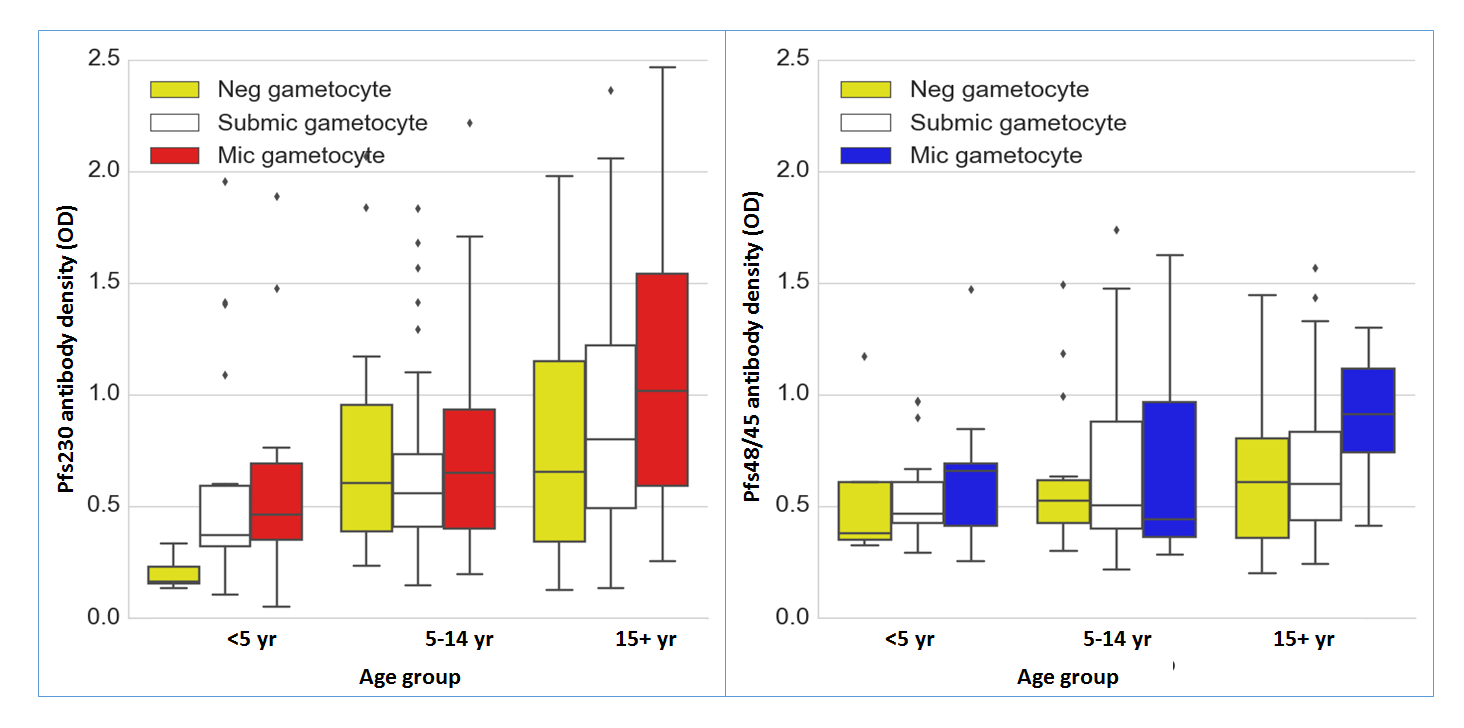

Supplement: S2 Fig — (TIF) [file ppat.1007034.s002.tif]

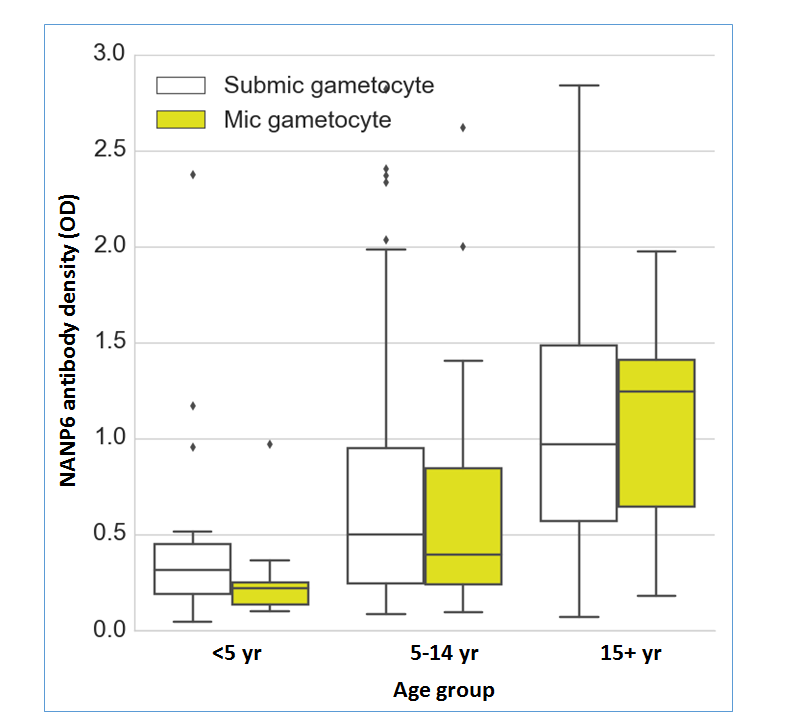

Supplement: S3 Fig — Antibody densities of study participants were measured in subsamples of n = 37, n = 97 and n = 157 samples in age groups 1–4, 5–14 and ≥15 years of age respectively. Error bars show horizontal lines from top indicating maximum antibody density, 75% percentile, median (50% percentile), 25% percentile and minimum antibody density. Dots outside shaded boxes represent outliers in the distribution of antibody densities within the given subsample. (TIF) [file ppat.1007034.s003.tif]
