# Supplementary material for: Modeling the impact of Plasmodium falciparum sexual stage immunity on the composition and dynamics of the human infectious reservoir for malaria in natural settings
Source: PLoS Pathog. 2018 May 9;14(5):e1007034. doi: 10.1371/journal.ppat.1007034 (PMC5962096; doi:10.1371/journal.ppat.1007034)
Supplement: S1 Appendix — (PDF) [file ppat.1007034.s004.pdf]

S1 appendix: supplemental methods

Section 1. Estimate of anti-Pfs230 and anti-Pfs48/45 antibody half-lives using a statistical model.

P. falciparum sexual stage immune model (Equation 2) was initialized with antibody half-lives determined in equation (1) using independent antibody data [1]. Equation (1) can be described as follow:

$$H = \left(\frac{\Delta\left(t_{i-j}\right)\ln(2)}{\log(Ai)-\log(Aj)}\right) \quad (1)$$ where:

H = Antibody half-life in day.

$\Delta(t_{i-j})$ = Duration between two consecutive sampling points i and j in days.

Ai = Median antibody titer at time point i for seropositive samples.

Aj = Median antibody titer at time point j for repeated samples as from time point i.

The method analyzes the change in antibody titers at the population level between the peak-wet (time point i) and end-wet (time point j) transmission season assuming alterations of antibody kinetics by frequent boosting (e.g. reinfection, persistent gametocyte carriage) unlikely.

The time-lag between sampling time points was estimated to be 111 days as from the last sampling day at the peak-wet season (August 23th) to the last sampling day at the end-wet season (December 12th).

For Pfs230, the antibody half-life was estimated 46.4 days (Median OD = 0.897 for n_i= 104 samples, Median OD = 0.258 for n_j = 24 samples). For Pfs48/45, the antibody half-life was estimated 142.0 days (Median OD = 0.8315 for n_i= 41, Median OD = 0.4837 for n_j = 10 samples).

***Section 2. Initialization and fitting procedures of P. falciparum* sexual stage immune model.**

Section 2.1 Model initialization

P. falciparum sexual stage immune model was initialized using simulated gametocyte data (Figure 1) as previously configured [2] and antibody half-lives as determined by a statistical model (section 1 - equation 1). The dynamical sexual stage immune model can be described as follow:

$$A(t_i) = A_0 e^{-ir} + \sum_{j=0}^{i-1} \beta(\gamma(t_{i-j}), d(t_{i-j})) e^{-jr} \quad (2)$$

where:
Equation (2) represents the antibody titer at any time (day) of life (t_i).
$A(t_i)$ = Antibody level at day t_i.
r = log (2)/antibody half-life = exponential antibody decay rate.
$\beta(\gamma(t_i), d(t_i))$ = Boost size at day t_i.
$\gamma(t_i)$ = gametocyte density at day t_i.
$d(t_i)$= Duration of exposure in days at day t_i.
A_0 = Initial antibody level from maternal immunity at day 0.

Section 2.2 Model fitting to estimate best anti-Pfs230 and anti-Pfs48/45 antibody half-lives.

P. falciparum sexual stage immune model was then fitted to Pfs48/45 and Pfs230 antibody data under the following structural assumptions: higher gametocyte density will boost more strongly than lower density – two categories were considered here (submicroscopic and microscopic gametocyte density) – and age-dependent exposure should impact the magnitude of the boost and the inter-individual variation within a smoothness constraint. Parameters were estimated by using observed features of antibody titers distributions: median titers and inter-quartile ranges by age, season, and current gametocyte density.

Multiple parameters sets were run to estimate the best fit allowing some stochasticity within a 95% confidence interval such that:

$$\beta(\gamma(t_i), d(t_i)) = \begin{cases} 0 \text{ if } i \leq 180 \\ \text{otherwise } \geq 0 \text{ with } \left| 1 - \frac{\frac{1}{n}\sum_{i=1}^{n} \alpha_i}{\frac{1}{n}\sum_{i=1}^{n} A(t_i)} \right| \leq \varepsilon \end{cases}$$ (3) where:

α_i and $A(t_i)$ = Antibody titers of individuals at day t_i from the field data and simulated data respectively, whose durations of exposure ($d(t_i)$) fall into the same bin.
ε (set to 0.1) = absolute difference (margin of error) between field data and simulated data when comparing means of antibody titers of individuals whose durations of exposure ($d(t_i)$) fall into the same bin.

Figure 1: Gametocyte prevalence from the present study (Data) and from simulation (EMOD malaria model [2])

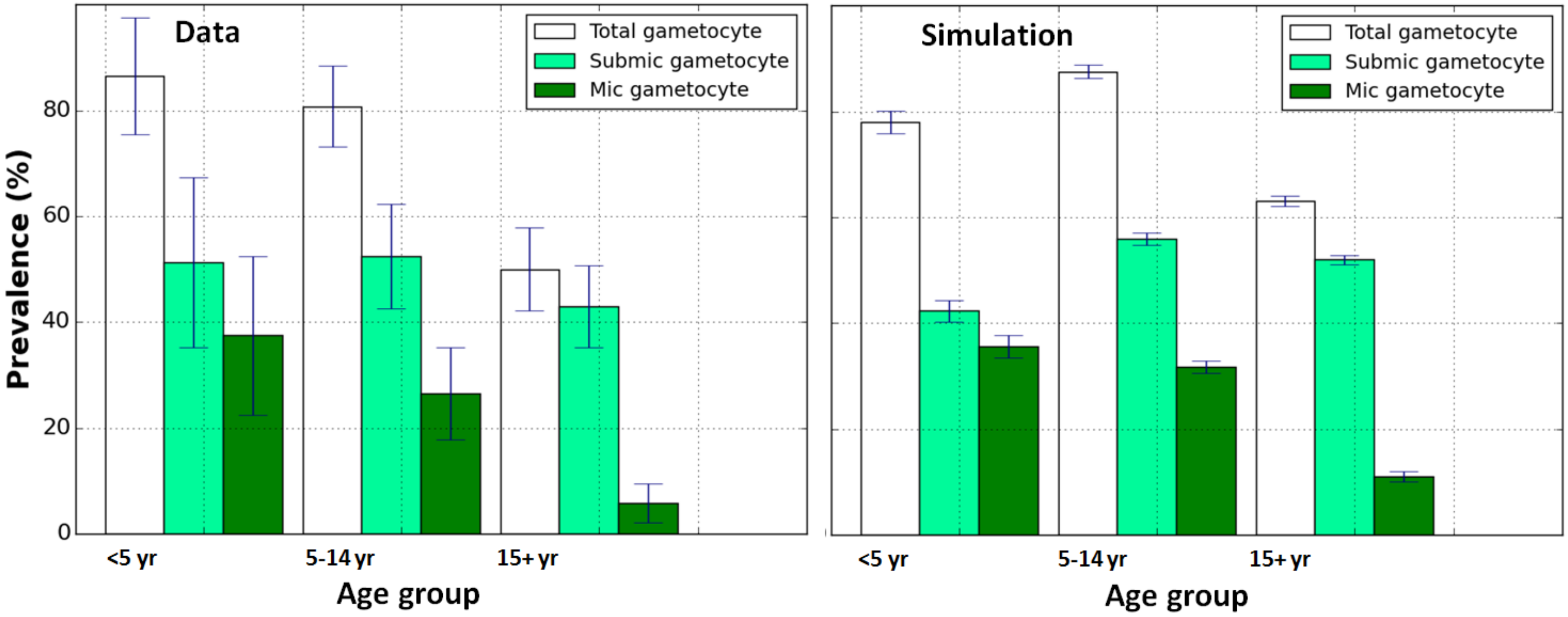

Section 2.2.1. Fitting outputs and interpretation

In simulation b (Table 1, Fig. 2), the model is initialized and run with Pfs230 antibody half-life equals to 46.4 days as determined in section 1 - equation (1). In simulation b, the model significantly diverges from the data likely due to slow antibody decay and / or over-estimation of boost sizes. In simulation c, the model is run as in b, except that boost size is reduced (half-lowered), which seems not to better fit the model.

In simulation d, the model is run as in c, except that the half-life of the antibody response is extended to 56 days. Note that antibody half-life was extended to 56 days by simply doubling our best fit antibody half-life of 28 days (simulation e). This part of the process aimed to test the impact of extended antibody half-life against lowered boost sizes. At first glance, an antibody half-life of 56 days well fits the model at the population level (Fig. 2 - 2d), except that the resolution in antibody response (median and inter-quartile ranges) between microscopic and submicroscopic gametocyte densities as observed in the field data is no picked up by the model (Fig. 2 – 1a versus Fig. 2 - 3d). In simulation e, the model seems to best fit the data balancing boost size and antibody half-life to account for fine resolution in immune boost and decay.

Two advantages were taken from the fitting procedure: i) Best estimates in boost sizes and antibody half-lives (28 days for both Pfs230 and Pfs48/45) fit the present data (S1 Fig) and data from and independent cohort study (26 and 32 days for Pfs230 and Pfs48/45 respectively, S1 Fig), ii) The present dynamical model estimates demonstrates that half-life of sexual stage antibodies can be as short as estimated at the population level (Equation (1), at least for anti-Pfs230 antibody half-life) and as reported in previous studies [3-6].

Table 1: Simulation outputs (Mean OD of antibody) under different parameters sets in comparison to data.

Data		Parameters set b® Half-life = 46.4 days			Parameters set c¥ Half-life = 46.4 days			Parameters set d¥ Half-life = 56 days			Parameters set e® Half-life = 28 days		
Age group	Mean OD	Boost size (β)*	Mean OD	δ (%)	Boost size (β)*	Mean OD	δ (%)	Boost size (β)*	Mean OD	δ (%)	Boost size (β)*	Mean OD	δ (%)
<5	0.5454	β0 = 0	0.7047	22.6	β0 = 0	0.4913	11.0	β0 = 0	0.5303	2.8	β0 = 0	0.4993	9.2
5-14	0.7053	β1 = 0.0085	0.9995	29.4	β1 = 0.0042	0.5456	29.2	β1 = 0.0042	0.6591	7.0	β1 = 0.0085	0.6267	12.5
15-29	0.8400	β2 = 0.0085	1.2842	34.5	β2 = 0.0042	0.7323	14.7	β2 = 0.0042	0.8639	2.7	β2 = 0.0085	0.8288	1.35
30+	0.8831	β3 = 0.0125	1.3711	35.6	β3 = 0.0062	0.7086	24.6	β3 = 0.0062	0.8622	2.4	β3 = 0.0125	0.8438	4.66
		β4 = 0.0125			β4 = 0.0062			β4 = 0.0062			β4 = 0.0125		
		β5 = 0.0175			β5 = 0.0087			β5 = 0.0087			β5 = 0.0175		
		β6 = 0.0175			β6 = 0.0087			β6 = 0.0087			β6 = 0.0175		
		β7 = 0.0310			β7 = 0.0155			β7 = 0.0155			β7 = 0.0310		

* Note that model fitting outputs incorporate boost size parameter for both microscopic and submicroscopic gametocyte densities. Only boost size for submicroscopic gametocyte density is presented in this table as an illustration. ® Simulation of the sexual stage immune model is done with boost size parameter values (best estimates) as presented in Table 2 and 3.

¥ Simulation of the sexual stage immune model is done with boost size parameter values (as presented in Table 2 and 3) multiplied by a factor of 0.5.

$\delta = \left| 1 - \frac{\frac{1}{n}\sum_{i=1}^{n} \alpha_i}{\frac{1}{n}\sum_{i=1}^{n} A(t_i)} \right|$ where δ is set to be ≤ ε.

Table 2. Model best parameter estimates for Pfs48/45 antibody responses assuming antibody half-life of 28 days

Parameter	Description	Time of exposure (Years)	Estimate	SD	Estimate	SD
γ	Gametocyte per µL blood		0.01-50		> 50	
α	Initial maternal antibody	0	0.6542	0.1	0.6542	0.1
β0	Boost	<0.5	0	0	0	0
β1	Boost	0.5-2	0.01	0.009	0.0275	0.025
β2	Boost	2-5	0.01	0.03	0.0275	0.025
β3	Boost	5-10	0.012	0.025	0.0165	0.15
β4	Boost	10-15	0.012	0.3	0.0165	0.325
β5	Boost	15-20	0.0175	0.015	0.0475	0.005
β6	Boost	20-30	0.0175	0.035	0.0475	0.005
β7	Boost	30+	0.0175	0.075	0.0475	0.045

Table 3. Model best parameter estimates for Pfs230 antibody responses assuming antibody half-life of 28 days

Parameter	Description	Time of exposure (Years)	Estimate	SD	Estimate	SD
γ	Gametocyte per µL blood		0.01-50		> 50	
α	Initial maternal antibody	0	0.7673	0.15	0.7673	0.15
β0	Boost	<0.5	0.0	0.0	0.0	0.0
β1	Boost	0.5-2	0.0085	0.01	0.0215	0.01
β2	Boost	2-5	0.0085	0.235	0.0215	0.235
β3	Boost	5-10	0.0125	0.070	0.0215	0.1
β4	Boost	10-15	0.0125	0.075	0.0215	0.13
β5	Boost	15-20	0.0175	0.085	0.08	0.215
β6	Boost	20-30	0.0175	0.105	0.08	0.29
β7	Boost	30+	0.0310	0.055	0.055	0.1

Figure 2: Fitting outputs (Mean OD of antibody) under different parameters sets in comparison to field data (a).
Median antibody titers from the study field data are shown in 1a and 2a. Simulations (b, c, d and e) are run with parameters sets (Table 1) to fit the data. Outputs 1, 2, 3 and 4 from each of simulations b, c, d and e represent: Antibody response dynamics at the individual level (1), mean antibody response at the population level (2), median antibody response per age at the population level under two gametocyte density categories (3) and median antibody response per season at the population level (4).

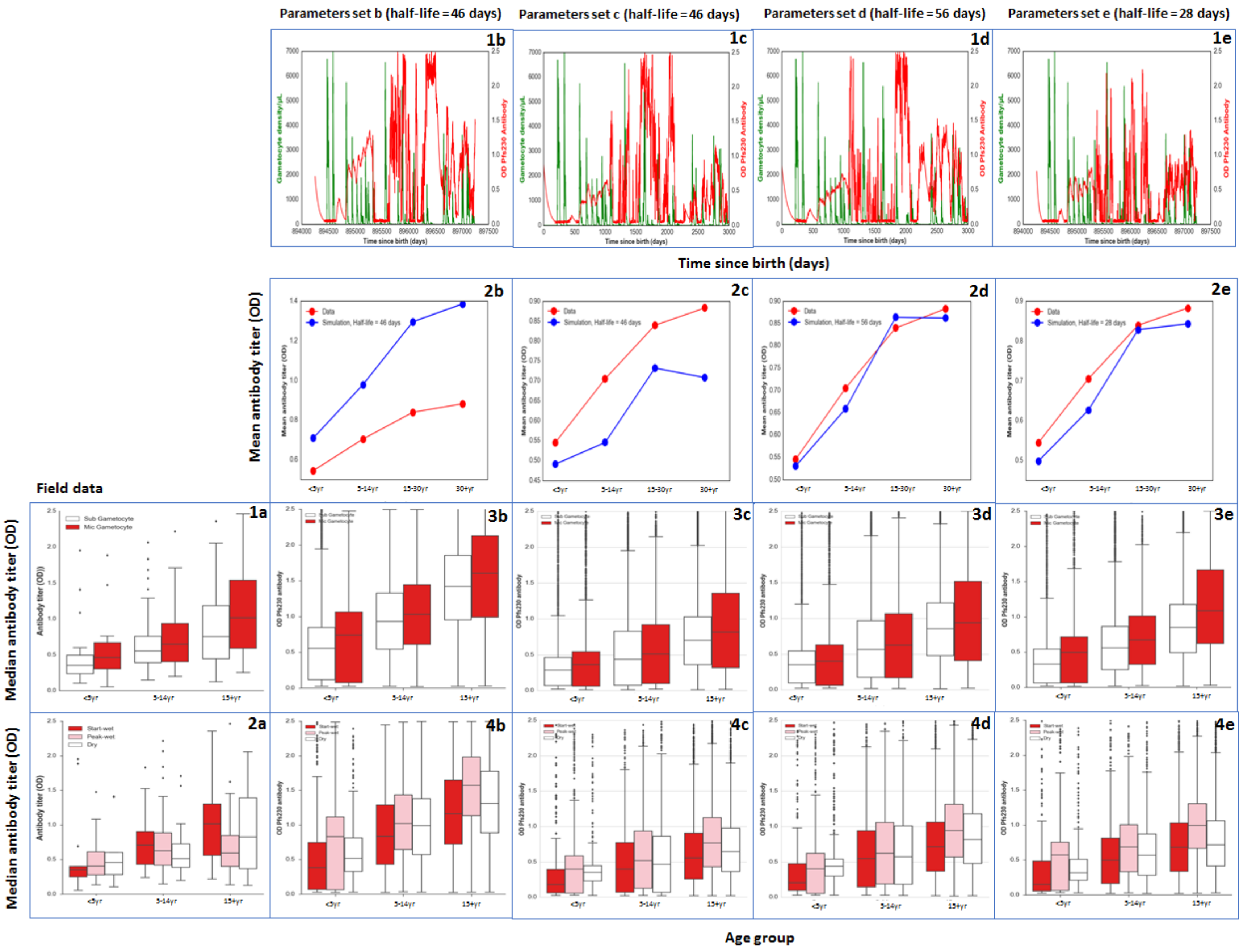

Parameters set b (half-life = 46 days)
Parameters set c (half-life = 46 days)
Parameters set d (half-life = 56 days)
Parameters set e (half-life = 28 days)
1b
1c
1d
1e
Gametocyte density/µL
OD Pfs230 Antibody
Time since birth (days)
2b
2c
2d
2e
Mean antibody titer (OD)
Data
Simulation, Half-life = 46 days
Simulation, Half-life = 56 days
Simulation, Half-life = 28 days
<5yr
5-14yr
15-30yr
30+yr
Field data
1a
3b
3c
3d
3e
Median antibody titer (OD)
Sub Gametocyte
Mic Gametocyte
Antibody titer (OD)
OD Pfs230 antibody
15+yr
2a
4b
4c
4d
4e
Start-wet
Peak-wet
Dry
Age group

Section 3. Oocyst simulation and effect of *P. falciparum* sexual stage immunity on transmission.

Regression coefficients (β values) that associate with anti-Pfs48/45 and anti-Pfs230 antibodies were transformed into odd ratios (e^{β}) and the effect of the corresponding antibody on oocyst intensity defined as:
$TRA_{oocyst} = 1 - e^{\beta}$ where TRA_{oocyst} is defined as transmission reducing activity on oocyst intensity.
The following TRA function:
$Y = Xe^{\beta}$ (3) where
Y = residual mean oocyst intensity
X = observed mean oocyst intensity,
$e^{\beta} = \text{Adjusted effect of immunity on oocyst intensity}$, was incorporated into the EMOD malaria model to assess the effect of sexual stage immunity on simulated oocyst intensity. Simulated oocyst intensity (X) per individual was sampled based on gametocyte density carriage using a kernel density estimation. Oocyst data from which are drawn the model's oocyst count (Fig 3) were obtained from individuals seronegative for both anti-Pfs48/45 and anti-Pfs230 antibodies in the field data and stratified by gametocyte density (submicroscopic, <16, 17-100, ≥100 gametocytes/µL).
Oocyst sampling was simulated 40 times in a closed loop mathematical draw to mimic a sample size of at least 20 fed mosquitoes required for the membrane feeding assay in the field. The arithmetic mean of oocyst intensity was then estimated per simulated individual. Residual oocyst intensity of a seropositive individual was obtained using equation (3) and the corresponding mosquito's infection rate was inferred using a polynomial interpolation over a reference curve [7] of the oocyst intensity versus oocyst prevalence as illustrated in Fig 4.
The reduction in mosquito's infection rate ($TRA_{mosquito}$) achieved in seropositive individuals was estimated using the following conventional TRA equation [7]:

$$\text{TRA}_{\text{mosquito}} = \frac{\text{Mean infection rate in Control group} - \text{Mean infection rate in Immune group}}{\text{Mean infection rate in Control group}} \text{x } 100 \quad (4)$$

Fig 3. Distribution of simulated mosquito's oocysts against measured field data.
N = 15000 observations from a birth cohort of 100 simulated individuals (over 50 years' simulation was performed to store data across all ages as in the field data) with oocyst intensity collected at different time points (start-wet, peak-wet and dry season).
Field data consisted of oocyst intensity collected at different time points (start-wet, peak-wet and dry season) from a sample size of N = 291 blood samples fed to mosquitoes.

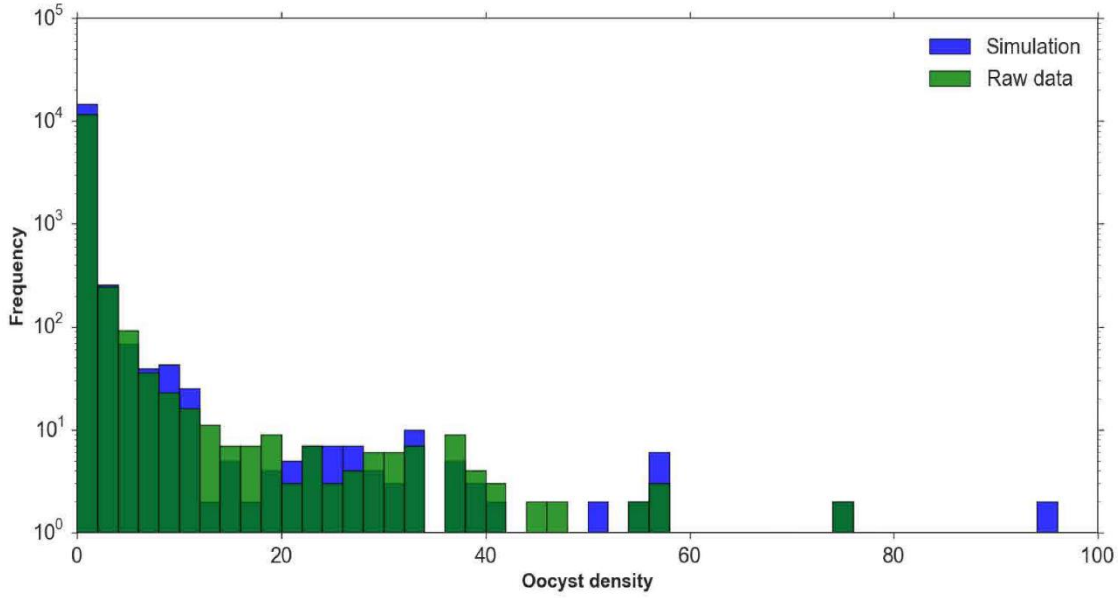

Figure 4: Illustration of the relationship between oocyst prevalence and intensity in standard membrane feeds data. Data extracted from [7].

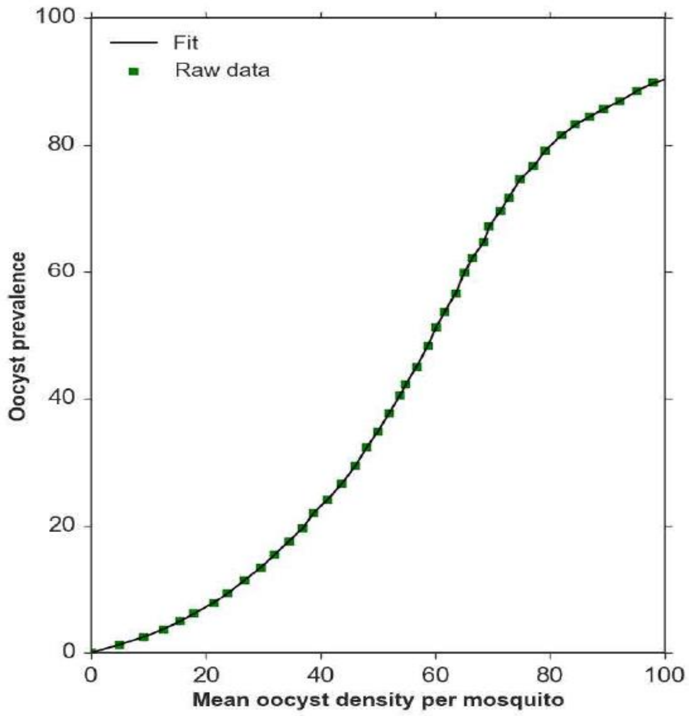

Section 4. Accounting for gametocyte density measurement uncertainty by QT-NASBA

Because we quantified gametocyte density by QT-NASBA, it appeared relevant that our simulations account for gametocyte measurement uncertainty by the QT-NASBA method. QT-NASBA is a RNA to DNA based pathogen detection method. The specificity of the assay [8] depends on primers that are specific for the RNA target. The reaction relies on enzymes AMV-RT, Rnase H and T7 RNA polymerase. An isothermal condition combined to T7 ensures that primers only anneal to single stranded target RNA and product cDNA preventing amplification of genomic DNA. Moreover, QT-NASBA uses a real-time molecular beacon technology combined to time to positivity (TTP) measurement as a proxy and a calibration curve of 10^0-10^6 gametocytes/μL dilution series to determine the parasite density in the test ample. Despite its high sensitivity and specificity, the method is subject to uncertainties because of random errors of analyzers (e.g. precision of TTP) or users (e.g. parallax type error). By averaging random error data extracted from a series of calibration curves [9], we established an uncertainty range within which our model's true gametocyte densities were randomly sampled, thus incorporating measurement uncertainty. Fig. 5 shows the 95% Confidence Interval of measurement uncertainty which increases with decreasing true gametocyte density.

When comparing to survey data quantified by QT-NASBA, the simulated true gametocyte densities were transformed by a log-normal smearing and fixed detection threshold (Fig. 5) to account for the method uncertainty described in [9].

Figure 5. Relationship between gametocyte density measurement uncertainty and simulated true gametocyte density.
N = 15000 observations from a birth cohort of 100 simulated individuals with data on gametocyte density collected at different time points (start-wet, peak-wet and dry season) over 50 years' simulation.

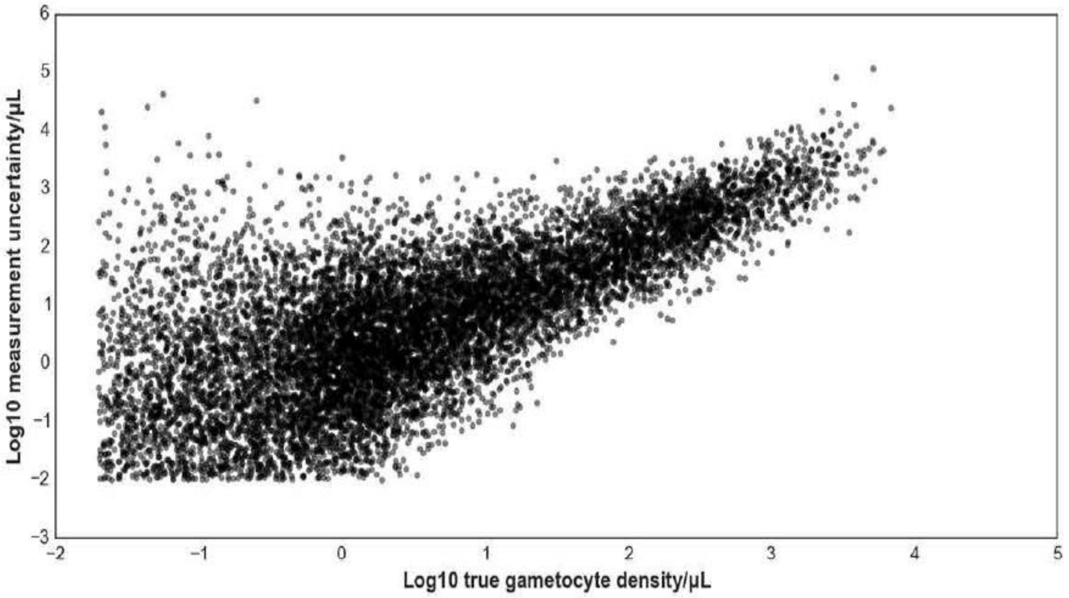

References

1. Ouedraogo AL, Roeffen W, Luty AJ, de Vlas SJ, Nebie I, Ilboudo-Sanogo E, et al. Naturally acquired immune responses to Plasmodium falciparum sexual stage antigens Pfs48/45 and Pfs230 in an area of seasonal transmission. Infection and immunity. 2011;79(12):4957-64. doi: 10.1128/IAI.05288-11. PubMed PMID: 21969000; PubMed Central PMCID: PMC3232645.
2. Gerardin J, Ouedraogo AL, McCarthy KA, Eckhoff PA, Wenger EA. Characterization of the infectious reservoir of malaria with an agent-based model calibrated to age-stratified parasite densities and infectiousness. Malaria journal. 2015;14:231. doi: 10.1186/s12936-015-0751-y. PubMed PMID: 26037226; PubMed Central PMCID: PMCPMC4702301.
3. Bousema T, Roeffen W, Meijerink H, Mwerinde H, Mwakalinga S, van Gemert GJ, et al. The dynamics of naturally acquired immune responses to Plasmodium falciparum sexual stage antigens Pfs230 & Pfs48/45 in a low endemic area in Tanzania. PloS one. 2010;5(11):e14114. doi: 10.1371/journal.pone.0014114. PubMed PMID: 21124765; PubMed Central PMCID: PMC2993929.
4. Bousema JT, Drakeley CJ, Kihonda J, Hendriks JC, Akim NI, Roeffen W, et al. A longitudinal study of immune responses to Plasmodium falciparum sexual stage antigens in Tanzanian adults. Parasite immunology. 2007;29(6):309-17. PubMed PMID: 17518949.
5. Bousema JT, Roeffen W, van der Kolk M, de Vlas SJ, van de Vegte-Bolmer M, Bangs MJ, et al. Rapid onset of transmission-reducing antibodies in javanese migrants exposed to malaria in papua, indonesia. The American journal of tropical medicine and hygiene. 2006;74(3):425-31. PubMed PMID: 16525101.
6. Wu Y, Ellis RD, Shaffer D, Fontes E, Malkin EM, Mahanty S, et al. Phase 1 trial of malaria transmission blocking vaccine candidates Pfs25 and Pvs25 formulated with montanide ISA 51. PloS one. 2008;3(7):e2636. PubMed PMID: 18612426.
7. Churcher TS, Blagborough AM, Delves M, Ramakrishnan C, Kapulu MC, Williams AR, et al. Measuring the blockade of malaria transmission--an analysis of the Standard Membrane Feeding Assay. International journal for parasitology. 2012;42(11):1037-44. doi: 10.1016/j.ijpara.2012.09.002. PubMed PMID: 23023048.
8. Compton J. Nucleic acid sequence-based amplification. Nature. 1991;350(6313):91-2.
9. Walker M, Basanez MG, Ouedraogo AL, Hermsen C, Bousema T, Churcher TS. Improving statistical inference on pathogen densities estimated by quantitative molecular methods: malaria gametocytaemia as a case study. BMC Bioinformatics. 2015;16:5. doi: 10.1186/s12859-014-0402-2. PubMed PMID: 25592782; PubMed Central PMCID: PMCPMC4307378.
